# Development and prospective validation of postoperative pain prediction from preoperative EHR data using attention-based set embeddings

**DOI:** 10.1038/s41746-023-00947-z

**Published:** 2023-11-16

**Authors:** Ran Liu, Rodrigo Gutiérrez, Rory V. Mather, Tom A. D. Stone, Laura A. Santa Cruz Mercado, Kishore Bharadwaj, Jasmine Johnson, Proloy Das, Gustavo Balanza, Ekenedilichukwu Uwanaka, Justin Sydloski, Andrew Chen, Mackenzie Hagood, Edward A. Bittner, Patrick L. Purdon

**Affiliations:** 1 Department of Anesthesia, Critical Care, and Pain Medicine, Massachusetts General Hospital, Boston, MA USA; 2 Harvard Medical School, Boston, MA USA; 3 Harvard-MIT Program in Health Sciences and Technology, Cambridge, MA US; 4 Department of Anesthesia, Critical Care, and Pain Medicine, Beth Israel Deaconess Medical Center, Boston, MA USA

**Keywords:** Medical research, Machine learning, Health care

## Abstract

Preoperative knowledge of expected postoperative pain can help guide perioperative pain management and focus interventions on patients with the greatest risk of acute pain. However, current methods for predicting postoperative pain require patient and clinician input or laborious manual chart review and often do not achieve sufficient performance. We use routinely collected electronic health record data from a multicenter dataset of 234,274 adult non-cardiac surgical patients to develop a machine learning method which predicts maximum pain scores on the day of surgery and four subsequent days and validate this method in a prospective cohort. Our method, POPS, is fully automated and relies only on data available prior to surgery, allowing application in all patients scheduled for or considering surgery. Here we report that POPS achieves state-of-the-art performance and outperforms clinician predictions on all postoperative days when predicting maximum pain on the 0–10 NRS in prospective validation, though with degraded calibration. POPS is interpretable, identifying comorbidities that significantly contribute to postoperative pain based on patient-specific context, which can assist clinicians in mitigating cases of acute pain.

## Introduction

Of the 51 million patients who undergo surgery each year in the United States^1^, as many as 80% experience acute postoperative pain^2,3^, and a majority report inadequate pain relief^3^. Almost 50% of patients report severe pain in the first 24 h of surgery^4^. Uncontrolled pain hinders postsurgical recovery, prolonging hospital stays, and increases mortality and the likelihood of chronic pain^5,6^. On the other hand, acute pain is commonly managed with opioids, prescribed to over 80% of surgical patients^1^; the risk of opioid use disorder, a present public health crisis^7,8^, increases with dosage and duration^1^. The American Pain Society recommends that clinicians individualize courses of treatment for each patient, yet existing assessments are heavily subjective, and many recommendations lack strong evidence^9^. Computational prediction of postoperative pain can provide quantitative guidance for perioperative pain management, and focus interventions on cases at greatest risk of acute pain

Existing literature on predicting postoperative pain is somewhat sparse and limited in scope. Only a handful of studies have attempted to compare pain across different procedure types^4^. Previous studies have used logistic regression to predict the likelihood of uncontrolled pain in relatively small cohorts of ambulatory^10^ and elective^11^ surgical cases, achieving moderate levels of performance. However, a major limitation of these studies is that they rely upon physician evaluations and patient surveys of anticipated pain^12,13^, and thus require human input and reflect the results of human prediction of postoperative pain more so than computational prediction.

In this study, we present a machine learning method, the Personalized post-Operative Pain prediction Score (POPS), for predicting postoperative pain in a wide range of surgeries using information about patients and procedures from commonly recorded preoperative electronic health record (EHR) data. We used neural networks to compute attention-based^14,15^ set embeddings from CPT and ICD-10 codes and use these embeddings in conjunction with structured EHR data to predict postoperative maximum pain scores on the day of surgery and four subsequent days. We developed POPS in a large, multicenter dataset. Furthermore, we validated the model in a prospective cohort and compared its performance against clinicians’ predictions.

## Results

**Fig. 1:**
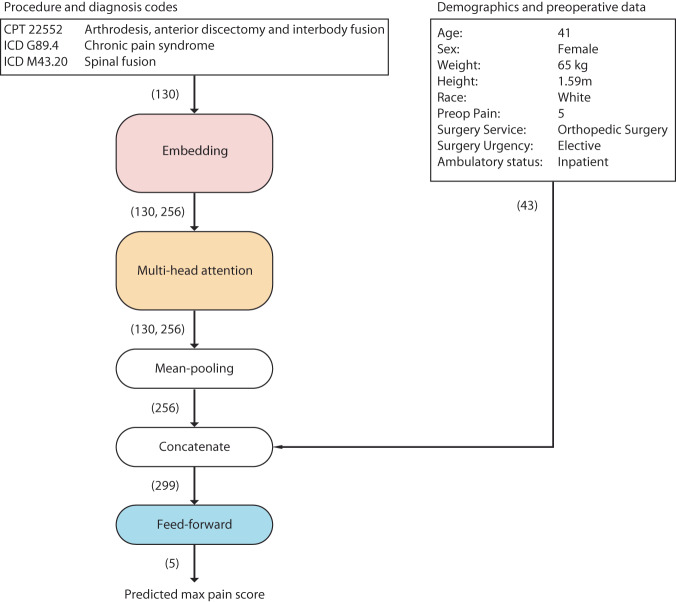
Structure of prediction model. Dimensionality of inputs, outputs, and intermediate representations are indicated in parentheses.

Our model consists of a neural network comprised of an embedding layer, multi-head self-attention layer^15^, and a densely connected feed-forward network (Fig. 1). For each patient, the network takes as input their set of CPT and ICD-10 codes, and computes a 256-dimensional vector, which we refer to as the set embedding. This set embedding is concatenated with a vector of demographic and preoperative variables and is then passed to the feed-forward network, which predicts the maximum pain score on the day of surgery and four subsequent postoperative days. We developed the prediction model using a multicenter retrospective dataset, and prospectively evaluated its performance. We also collected clinician predictions of postoperative pain for surgical cases in the prospective cohort and compared the performance of our model’s predictions against clinician predictions.

### Study cohorts and datasets

Our retrospective dataset consists of preoperative electronic health record data collected for 234,274 adult patients who underwent surgery between April 1^st^, 2016, and March 31^st^, 2020, across four hospitals: two quaternary care academic medical centers, Massachusetts General Hospital (MGH) and Brigham and Women’s Hospital (BWH], and two community hospitals, North Shore Medical Center (NSMC) and Newton Wellesley Hospital (NWH). Baseline demographics of the retrospective cohort encompassing surgical patients from these four hospitals are presented in Table 1. We included all adult non-cardiac surgical cases with general anesthesia (inpatient, outpatient, urgent, emergent, elective) and at least one recorded pain score on the day of surgery and four subsequent days. Of the patients in our retrospective study cohort, 130,713 (55.79%) were women; 192,664 (82.24%) were White non-Hispanic. The mean age was 55.9 years (SD 17.0). Orthopedic, general, urological, gynecological, and thoracic surgeries were the most common, comprising a combined 66.8% of surgeries. In the retrospective cohort, 40 patients were excluded because they died during surgery, and 15,853 were excluded because they were admitted to the intensive care unit immediately after surgery.

**Table 1:** Baseline statistics for the retrospective and prospective cohort.

Statistic		Retrospective	(%)	Prospective	(%)	*p*-value
Total patients		234,274	(100.0)	365	(100.00)	
MGH		104,972	(44.81)	365	(100.00)	
BWH		85,524	(36.51)	0	(0.00)	
NSMC		30,720	(13.11)	0	(0.00)	
NWH		13,058	(5.57)	0	(0.00)	
Demographics						
Age, mean (SD) years		55.9	(17.0)	59.6	(16.0)	<0.001
Sex						0.0934
	Male	103,554	(44.20)	182	(49.86)	
	Female	130,713	(55.79)	183	(50.14)	
Race						0.212
	White	192,664	(82.24)	297	(81.37)	
	Black	11,822	(5.05)	13	(3.56)	
	Hispanic	9301	(3.97)	21	(5.75)	
	Asian	7173	(3.06)	9	(2.47)	
	Other	13,314	(5.68)	25	(6.85)	
Height, mean (SD) m		1.69	(0.11)	1.69	(0.12)	0.373
Weight, mean (SD) kg		81.42	(24.46)	83.38	(22.95)	0.105
Opioid naivety		163,971	(69.99)	236	(64.66)	0.0304
Clinical characteristics						
ASA						<0.001
	I	23,133	(9.87)	10	(2.74)	
	II	113,778	(48.57)	132	(36.16)	
	III	90,509	(38.63)	215	(58.90)	
	IV	6483	(2.77)	7	(1.92)	
Ambulatory surgery		95,574	(40.80)	0	(0.00)	
Inpatient surgery		138,696	(59.20)	365	(100.00)	
Elixhauser comorbidity index, median (IQR)		0.0	(0.0, 5.0)	0.0	(0.0, 5.0)	0.549
Surgical service						<0.001
Orthopedic Surgery		57,027	(24.34)	62	(16.99)	
General Surgery		35,023	(14.95)	104	(28.49)	
Urology		24,500	(10.46)	32	(8.77)	
Gynecology		22,316	(9.53)	10	(2.74)	
Thoracic Surgery		17,586	(7.51)	55	(15.07)	
Neurosurgery		16,596	(7.08)	63	(17.26)	
Surgical Oncology		13,715	(5.85)	0	(0.00)	
Other		47,511	(20.28)	39	(10.68)	

*MGH* Massachusetts General Hospital, *BWH* Brigham and Women’s Hospital, *NSMC* North Shore Medical Center, *NWH* Newton-Wellesley Hospital, *ASA* American Society of Anesthesiologists Physical Status Classification System.

*P* values are reported for two-sided tests of differences in distributions of variables between the retrospective and prospective cohorts. Differences in distributions of categorical variables (sex, race, opioid naivety, ASA status, surgical service) between the retrospective and prospective cohorts were assessed using the Chi-squared test. Differences in distributions of normally distributed random variables (age, height, weight) were assessed using the t-test.

We conducted a prospective study at Massachusetts General Hospital, in which 365 adult non-cardiac surgical patients were enrolled between February 15^th^, 2023, and March 20^th^, 2023. Baseline demographics of the prospective cohort are presented in Table 2. 183 (50.1%) were women (as a biological attribute – sex); 297 (81.4%) were White non-Hispanic. The mean age was 59.6 years (SD 16.0). General, neurosurgery, orthopedic, and thoracic surgeries were the most common, comprising a combined 77.8% of surgeries. Clinicians from the anesthesia team were surveyed at the beginning of each case, and their predictions on expected postoperative pain recorded (Supplementary Methods). These outcomes were also predicted using our model developed on the retrospective dataset. Only inpatients were included in the prospective study to allow evaluation of maximum pain score outcomes beyond the day of surgery. The number of patients with at least one recorded pain score, with moderate or severe pain, and the number of patients excluded from evaluation on each postoperative day is given in Supplementary Table 1. In the prospective cohort, 20 patients were excluded because they were admitted to the intensive care unit immediately after surgery; none died during surgery.

**Table 2:** Example patients derived from clinical gestalt and resulting model predictions of postoperative pain.

Characteristic	Patient 1	Patient 2	Patient 3
Age (years)	41	74	58
Sex	Female	Male	Female
Weight (kg)	65	74	79
Height (m)	1.59	1.68	1.55
Race	White	Black	Asian
Preop Pain Score	5	0	2
Surgery Service	Orthopedic Surgery	Urology	Otolaryngology
Surgery Urgency	Elective	Elective	Non-urgent
Inpatient/Outpatient	Inpatient	Outpatient	Inpatient
Surgery (CPT)	Arthrodesis, anterior discectomy and interbody fusion (22552)	Cystoscopy (52260)	Surgical nasal/sinus endoscopy (31255)
Chronic pain (G89.4)	X		
Tobacco use (Z72.0)	X	X	
Fibromyalgia (M79.7)			X
Sleep disorder (G47.9)	X		
Depression (F32.A)			X
Spinal fusion (M43.20)	X		
Opioid dependence (F11.29)		X	
Hypercholesterolemia (E78.00)		X	
Hypertension (I10)		X	
Cyst and mucocele of nose and nasal sinus (J34.1)			X
Hypothyroidism (E03.9)			X
Predicted Max Pain (Postop Day 0)	7.93 (7.09, 8.64)	2.51 (1.61, 3.45)	6.38 (5.58, 7.14)
Predicted Max Pain (Postop Day 1)	7.88 (7.10, 8.68)	0.67 (0.00, 1.59)	5.69 (4.86, 6.57)
Predicted Max Pain (Postop Day 2)	7.37 (6.63, 8.14)	1.17 (0.17, 2.19)	5.25 (4.48, 6.13)
Predicted Max Pain (Postop Day 3)	7.19 (6.35, 8.01)	0.92 (0.00, 1.96)	5.13 (4.27, 6.02)
Predicted Max Pain (Postop Day 4)	7.17 (6.17, 8.12)	1.27 (0.27, 2.29)	4.96 (4.07, 5.90)

*CPT* Current Procedural Terminology.

Pain scores in the EHR were recorded numerically, or in text form. In our retrospective cohort, 222,374 out of 234,274 patients (94.9%) have at least one numeric pain score; 91,270 (39.0%) have pain strings. Overall, pain strings comprise 432,042 out of 4,925,886 recorded pain scores (8.8%). In our prospective cohort, all 365 patients (100%) have numeric pain scores; 130 (35.6%) have pain strings, and pain strings comprise 541 out of 11,100 recorded pain scores (4.9%). The distribution of recorded pain scores by type is shown in Supplementary Fig. 1.

The most frequently observed CPT and ICD-10 codes in our study cohorts are reported in Supplementary Tables 2–5. The overall distribution of pain outcomes in the retrospective and prospective cohorts is shown in Supplementary Fig. 2. In the retrospective cohort, the mean maximum pain score on each day was 5.2 on Postoperative Day 0, 5.3 on Postoperative Day 1, 5.5 on Postoperative Day 2, 5.2 on Postoperative Day 3, and 5.2 on Postoperative Day 4. In the prospective cohort, the mean maximum pain score on each day was 6.6 on Postoperative Day 0, 6.4 on Postoperative Day 1, 6.1 on Postoperative Day 2, 5.7 on Postoperative Day 3, and 5.5 on Postoperative Day 4. The retrospective and prospective cohorts significantly differ in distributions of outcomes on postoperative days 0–2.

The distribution of the number of pain score observations per patient on each postoperative day is shown in Supplementary Fig. 3. Outcomes are only available for patients who are present in the hospital on each postoperative day, and so on later days, outcomes are conditioned on patients’ length of hospital stay. The distribution of outcomes for patients grouped by the day of their last observed pain score is shown in Supplementary Fig. 4. Average pain scores within each subgroup are decreasing, but patients with longer stays had higher average pain trajectories. Supplementary Tables 6–10 report baseline statistics for the subgroups of patients not excluded on each postoperative day. Patients present on later days were on average older, had more comorbidities, higher ASA scores, and were less likely to be opioid naive.

### Retrospective prediction of postoperative pain

**Fig. 2:**
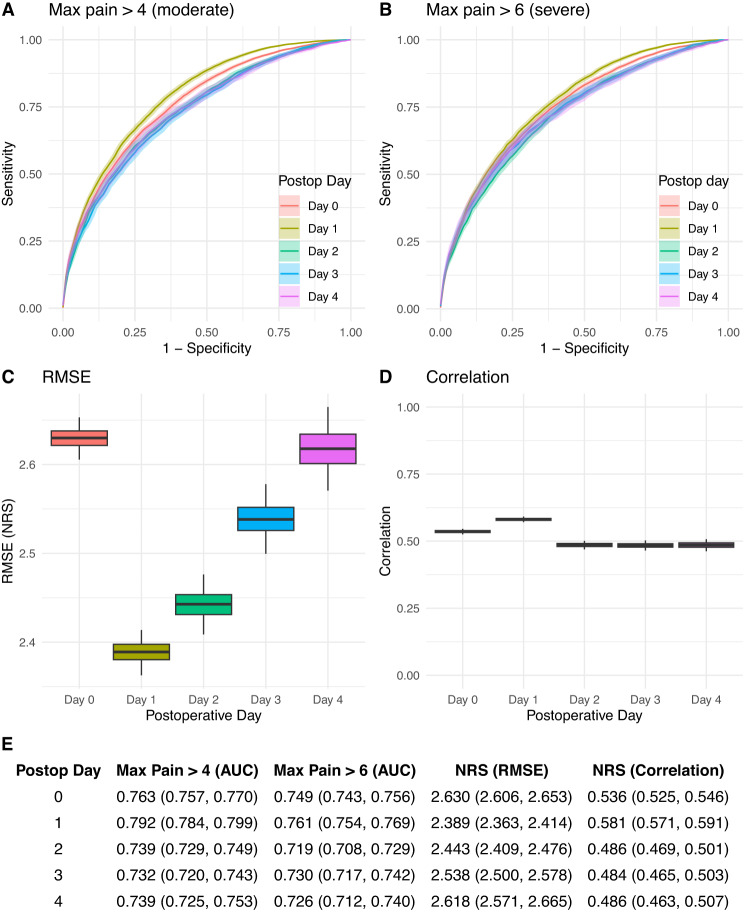
Performance metrics for prediction of postoperative pain in the retrospective cohort. Performance was assessed by AUC for NRS > 4 (**A**) and >6 (**B**), RMSE (**C**), and correlation (**D**) for POPS, along with a table of metrics (**E**). Shaded areas, error bars, and values represent ±1 standard deviation. Colors indicate different postoperative days. Boxplots (**C**, **D**) represent the median (central line), interquartile range (bounds of box) and 1 standard deviation (whiskers). RMSE Root-mean square error, NRS Numeric Rating Scale, AUC area under the curve.

In our retrospective cohort, our model achieves moderate performance in predicting moderate (Fig. 2A, defined as a maximal pain score above 4 on the 0–10 numeric rating scale (NRS)^16^) and severe pain (Fig. 2B, defined as a maximal pain score above 6 on the NRS). Area under the receiver operating curve (AUC) ranged between 0.73 and 0.79 on postoperative days 0 through 4 for predictions of moderate pain, and between 0.72 and 0.76 for predictions of severe pain. We also computed performance within each of the 10 most frequent surgical services (Supplementary Tables 11–14). Similar performance was achieved across services, with best performance in Otolaryngology and Neurosurgery, and slightly poorer performance in Urology and Gynecology.

When predicting expected maximum pain on the NRS as a continuous variable, our model yields a root mean squared error (RMSE) between 2.39 and 2.63 points (Fig. 2C), and a Pearson correlation coefficient between 0.49 and 0.58 (Fig. 2D). The best predictive performance across all reported measures was achieved on postoperative day 1. Good calibration in the retrospective cohort was observed for all predictions (Supplementary Fig. 5).

For each hospital in the retrospective cohort, we also trained models using data from only one hospital and evaluated predictions of postoperative pain for those models on test data from the remaining three sites (Supplementary Tables 15–18). We find that our fitted models are relatively robust when predicting pain for patients from sites unseen during training with little to no degradation of performance on most postoperative days.

### Prospective prediction of postoperative pain

**Fig. 3:**
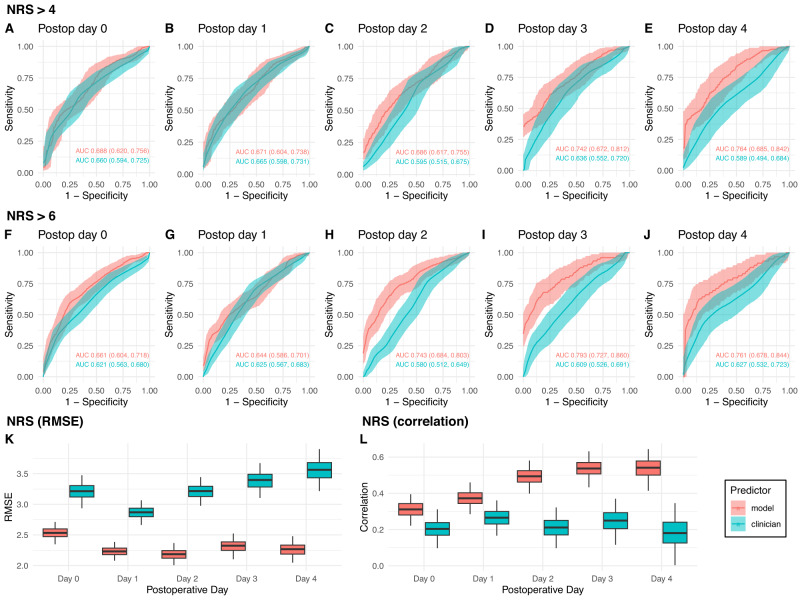
Performance metrics for prediction of postoperative pain in the prospective cohort. For POPS and clinician predictions, we present Receiver Operating Curves (ROCs) for NRS > 4 and >6 with Area Under the Curve (AUC) (**A**–**J**), RMSE (**K**), and correlation (**L**). Shaded areas, error bars, and values represent ±1 standard deviation. Boxplots (**K**, **L**) indicate the median (central line), interquartile range (bounds of box) and 1 standard deviation (whiskers). RMSE Root-mean square error, NRS Numeric Rating Scale. Red represents POPS while cyan indicate clinician performance.

In our prospective cohort, POPS predicts postoperative pain with performance comparable to that achieved in the retrospective data, though with poorer performance on postoperative days 0 and 1. AUCs ranged from 0.67 to 0.76 on postoperative days 0 through 4 for predictions of moderate pain (Fig. 3A–E), and between 0.64 and 0.79 (Fig. 3F–J) for predictions of severe pain. When predicting expected maximum pain on the NRS as a continuous variable, POPS achieves RMSEs of 2.19 to 2.53 points (Fig. 3K) and correlations between 0.31 and 0.54 (Fig. 3L). Calibration plots showed some deviation between expected and observed postoperative pain levels for predictions made by POPS in the prospective cohort (Supplementary Fig. 6), and poor calibration for predictions made by clinicians (Supplementary Fig. 7). Supplementary Tables 19–22 report observed/expected ratios for binarized outcomes, and calibration intercept and slope. We also evaluated the performance of all single-center models on our prospective cohort (Supplementary Tables 23–26). We also computed the total dosage of intraoperative opioid administration in both cohorts. Patients in the prospective cohort received more hydromorphone on average than patients in the retrospective cohort (Supplementary Fig. 8).

### POPS outperforms clinicians at predicting postoperative pain

Clinicians surveyed at the beginning of each surgical case in the prospective cohort achieved AUCs of 0.59 to 0.66 on postoperative days 0 through 4 for predictions of moderate pain (Fig. 3A–E) and between 0.58 and 0.63 (Fig. 3F–J) for predictions of severe pain. POPS achieved significantly better AUCs on postoperative days 2–4. When predicting expected maximum pain on the NRS as a continuous variable, clinician predictions have RMSEs of 2.87 to 3.56 points (Fig. 3K) and correlations between 0.18 and 0.27 (Fig. 3L). POPS achieved significantly better performance than clinicians by RMSE and correlation on all days.

### Hypothetical example patients derived from clinical knowledge

We defined three hypothetical example patients with demographic information, preoperative variables, and CPT and ICD codes required to compute the POPS (Table 3). Patient information was outlined by an anesthesiologist who aimed to characterize three archetypical subjects with different expected postoperative pain profiles based on existing clinical knowledge. These example patients illustrate the type of data required to compute POPS and serve as an assessment of face validity.

**Table 3:** Attention weights and impact of each CPT or ICD-10 code on postoperative pain predictions made by POPS for each of the three example patients in Table 3.

Description	Attention Weight	ΔDay 0	ΔDay 1	ΔDay 2	ΔDay 3	ΔDay 4
Patient 1		7.93 (7.09, 8.64)	7.88 (7.10, 8.68)	7.37 (6.63, 8.14)	7.19 (6.35, 8.01)	7.17 (6.17, 8.12)
ICD G89.4 (Chronic pain syndrome)	0.0103	**0.36 (0.11, 0.71)**	**0.42 (0.15, 0.77)**	**0.37 (0.09, 0.71)**	**0.38 (0.10, 0.72)**	**0.37 (0.06, 0.72)**
ICD M43.20 (Fusion of spine, site unspecified)	0.0089	−0.03 (−0.37, 0.25)	−0.04 (−0.42, 0.23)	−0.04 (−0.36, 0.24)	−0.03 (−0.39, 0.22)	−0.03 (−0.40, 0.21)
CPT 22552 (Arthrodesis ant interbody inc discectomy, cervical below c2 each addl)	0.0088	0.08 (−0.23, 0.40)	0.10 (−0.22, 0.42)	0.09 (−0.20, 0.37)	0.09 (−0.22, 0.38)	0.08 (−0.20, 0.42)
ICD Z72.0 (Tobacco use)	0.0088	0.08 (−0.13, 0.30)	0.10 (−0.15, 0.36)	0.08 (−0.15, 0.32)	0.08 (−0.18, 0.30)	0.07 (−0.16, 0.31)
ICD G47.9 (Sleep disorder, unspecified)	0.0088	0.09 (−0.11, 0.34)	0.12 (−0.09, 0.35)	0.09 (−0.12, 0.32)	0.10 (−0.13, 0.35)	0.10 (−0.14, 0.37)
Patient 2		2.51 (1.61, 3.45)	0.67 (0.00, 1.59)	1.17 (0.17, 2.19)	0.92 (0.00, 1.96)	1.27 (0.27, 2.29)
ICD F11.29 (Opioid dependence with unspecified opioid−induced disorder)	0.0100	−0.03 (−0.45, 0.41)	−0.03 (−0.45, 0.36)	−0.03 (−0.54, 0.44)	−0.03 (−0.53, 0.39)	−0.01 (−0.47, 0.40)
CPT 52260 (Cystoscopy, dil bladder, gen anesthesia)	0.0099	−0.09 (−0.55, 0.33)	−0.10 (−0.50, 0.28)	−0.12 (−0.60, 0.32)	−0.11 (−0.54, 0.31)	−0.09 (−0.52, 0.32)
ICD Z72.0 (Tobacco use)	0.0096	0.04 (−0.31, 0.36)	0.01 (−0.34, 0.31)	−0.02 (−0.39, 0.32)	−0.02 (−0.38, 0.33)	−0.01 (−0.32, 0.31)
ICD E78.00 (Pure hypercholesterolemia, unspecified)	0.0079	−0.02 (−0.24, 0.17)	−0.00 (−0.19, 0.18)	−0.04 (−0.27, 0.17)	−0.03 (−0.23, 0.18)	−0.03 (−0.25, 0.19)
ICD I10 (Essential (primary) hypertension)	0.0069	−0.04 (−0.26, 0.12)	−0.06 (−0.25, 0.12)	−0.06 (−0.27, 0.13)	−0.06 (−0.25, 0.13)	−0.07 (−0.27, 0.12)
Patient 3		6.38 (5.58, 7.14)	5.69 (4.86, 6.57)	5.25 (4.48, 6.13)	5.13 (4.27, 6.02)	4.96 (4.07, 5.90)
ICD M79.7 (Fibromyalgia)	0.0095	**0.28 (0.05, 0.54)**	**0.28 (0.03, 0.57)**	**0.26 (0.02, 0.58)**	**0.28 (0.02, 0.57)**	0.26 (−0.04, 0.57)
CPT 31255 (Nasal/sinus endoscopy, remv totl ethmoid)	0.0093	−0.00 (−0.34, 0.29)	0.02 (−0.36, 0.40)	0.03 (−0.35, 0.39)	0.02 (−0.38, 0.40)	0.01 (−0.39, 0.38)
ICD J34.1 (Cyst and mucocele of nose and nasal sinus)	0.0092	0.08 (−0.23, 0.42)	0.09 (−0.26, 0.49)	0.08 (−0.25, 0.49)	0.08 (−0.25, 0.49)	0.08 (−0.27, 0.52)
ICD E03.9 (Hypothyroidism, unspecified)	0.0079	0.02 (−0.14, 0.18)	0.01 (−0.17, 0.18)	−0.01 (−0.20, 0.18)	0.02 (−0.17, 0.21)	0.01 (−0.20, 0.22)
ICD F32.A (Depression, unspecified)	0.0077	0.12 (−0.02, 0.28)	**0.20 (0.05, 0.38)**	**0.18 (0.01, 0.36)**	**0.19 (0.03, 0.40)**	0.19 (−0.00, 0.40)

*ICD* International Statistical Classification of Diseases and Related Health Problems.

95% confidence intervals are given in parentheses. Impacts of CPT or ICD-10 codes on predicted postoperative pain whose confidence intervals do not cross zero are indicated in bold. ICD: International Statistical Classification of Diseases and Related Health Problems.

Patient 1 represents a patient with many known risk factors for postoperative pain undergoing a relatively painful spinal surgery. Accordingly, POPS predicts high expected maximum NRS pain scores ranging from 7.93 on the day of surgery to 7.17 on the fourth postoperative day.

Patient 2 has no known risk factors and is undergoing a minor elective urological procedure. POPS predicts a maximum pain score of 2.51 on the day of the surgery, then a maximum pain score of around 1 on all subsequent days.

Patient 3 represents an intermediate example with fewer risk factors than Patient 1. POPS predicts a maximum pain score of 6.38 on the day of surgery, decreasing to 4.96 by the fourth postoperative day.

### Attention weights identify comorbidities which impact pain

Table 4 reports attention weights computed by the multi-head self-attention layer of the neural network, along with the estimated effect of each CPT or ICD-10 code on the predicted postoperative pain of each patient and bootstrapped confidence intervals. Attention weights indicate the relative importance of each code in terms of contribution to predicted postoperative pain levels; Patient 1’s diagnosis of chronic pain syndrome is identified by POPS as their most impactful comorbidity, associated with an increase of 0.36 to 0.42 points on the NRS in expected maximum pain score on each postoperative day compared to an identical patient without that specific diagnosis. The next most impactful codes are their spinal fusion, for which they are undergoing surgery, and the CPT for their arthrodesis with discectomy, followed by their history of tobacco use and sleep disorder, with similar attention weights for all four indicating that these codes are roughly equal in importance. In Patient 2, their most important comorbidity is their history of opioid dependence, though the direction of effect is not significantly known, whereas their least important comorbidities are hypercholesterolemia and hypertension. In Patient 3, their diagnosis of fibromyalgia is identified by attention weights as their most impactful comorbidity, associated with significant increases in expected maximum pain score on postoperative days 0 through 3 of 0.26 to 0.28 points.

**Table 4:** Comparison of POPS against methods found in our literature review of preoperative prediction of postoperative pain.

Study	Retrospective cohort size	Prospective cohort size	Patient interaction	Distribution of surgical services	Outcome	Performance
POPS (ours)	243,274	365	No	Orthopedic: 24.3% General: 15.0% Urology: 10.5% Gynecology: 9.5% Thoracic: 7.5% Neurosurg.: 7.1% Surg. Oncology: 5.9% Other: 20.3%	Moderate and severe pain on postop days 0–4	0.72–0.79 AUC
Armstrong et al. 2023	17,079		Yes	General: 62.0% Urology: 15.6% Thoracic: 7.5% Orthopedic: 5.5% Neurosurg.: 3.1% Gynecology: 1.7% Vascular: 0.7% Other: 3.9%	Severe pain on first postop day	0.66 optimism-corrected c-statistic
Van Driel et al. 2022	344	150	Yes	Orthopedic: 45.3% General: 20.9% Vascular: 19.5% Other: 14.2%	Persistent post-surgical pain at 3 months	0.7 AUC
Rehberg et al. 2017		198	Yes	Surg. Oncology: 100%, only breast cancer surgeries	Max pain on first postop day	0.82 AUC
Hur et al. 2021	112,989		No	Unreported, 13 common procedures	30-day refill, new persistent use	0.66–0.68 AUC
Janseen et al. 2008	549	1035	Yes	Unreported, non-cardiac surgery	Severe pain 1 h after surgery	0.65 AUC
Kallman et al. 2003		1416	Yes	Unreported, non-cardiac surgery	Severe pain 1 h after surgery	0.73 AUC
Sommer et al. 2010		1490	No	Unreported, non-cardiac surgery	Mean pain (VAS) > 4	0.74–0.78 AUC

*AUC* Area under the receiver operating curve.

## Discussion

In this study, we present POPS, a method for computational prediction of postoperative pain score using routinely recorded preoperative EHR data. We developed our model using a large, multicenter dataset which includes two quaternary care academic medical centers, MGH and BWH, and two community hospitals, NSMC and NWH. We demonstrated its predictive performance and showed that it can reasonably generalize between hospitals within our retrospective cohort.

We prospectively validated POPS at MGH and compared our model’s predictions of postoperative pain to those made by clinicians. In predicting moderate or severe pain, our model performed similarly to clinicians on postoperative days 0 and 1, and outperformed clinicians by postoperative days 2–4. In predicting pain on the 0–10 NRS, our model outperformed clinicians on all days.

POPS relies solely upon variables from the electronic health record which are available prior to surgery without the need for clinician assessment, manual chart review, or patient input, and thus can be potentially applied in all patients scheduled for or considering surgery without increasing clinician workload.

Predictions provided by our model may help clinicians improve perioperative pain management. Knowledge of expected postoperative pain levels provided by POPS could be used by the anesthesia care team to identify patients who could benefit from a more detailed pre-operative pain management workup, such as obtaining a more detailed history of past opioid usage, chronic pain diagnoses, or other predictors of increased postoperative pain. It could also be used to inform analgesic strategies within the operating room, such as the use of long-acting opioids during surgery so that intraoperative analgesic coverage extends into postoperative recovery^17^, or the use of regional analgesia^18,19^. Nonetheless, numerical pain scores do not perfectly reflect the need for pain management and should not be the sole basis for clinician decision-making^20,21^. In that sense, POPS is a tool that may aid planning a priori but must be combined with clinician evaluations of each individual patient.

Information provided by POPS may also help establish realistic expectations in terms of postoperative pain with patients^22^, and facilitate improved joint decision making by patients and clinicians in the perioperative period^23^. In recent work, we found that some intraoperative opioid administration leads to better short and long-term pain-related outcomes after surgery^24^. However, it remains unclear how much opioid administration is optimal at an individual level. Individualization of pain management using predicted postoperative pain may mean less intraoperative opioid administration for patients with low predicted pain, and greater intraoperative opioid usage for patients with higher predicted pain. Future studies may evaluate whether the implementation of this score can lead to reductions in acute postoperative pain, reductions in postoperative opioid administration, or improvements to patient satisfaction and quality of recovery.

Our model is interpretable and can identify the most important ICD-10 and CPT codes within each patient’s specific context, as well as their effects upon postoperative pain. This may aid in personalization of pain management strategies. In example Patient 3, our model identified the most informative codes as their history of fibromyalgia, their presenting diagnosis of a nasal cyst, and the scheduled removal procedure (Table 4). For instance, it has been well established that fibromyalgia is associated with worse postoperative pain^25^ and more opioid requirements^26,27^. For patient 1, the presence of preoperative chronic pain diagnosis was a clear driver of a higher postoperative pain predicted by the model, which is in agreement with previous studies^28,29^. In addition, the age and sex of this patient are known risk factors for greater postoperative pain^29^.

The majority of existing studies (Table 4) on predicting postoperative pain have been conducted on relatively small cohorts^13,30–32^ and lack external validation^10,33–35^, which limits their generalizability. Recent concerns have been raised regarding a lack of methodological rigor in the development of clinical risk predictors^36^. Furthermore, these models often predict postoperative pain for only one specific type of surgery, rather than for a general surgical population, which renders their usage in clinical practice impractical.

Recently, Armstrong et al. conducted a study using logistic regression on preoperative variables to predict severe pain after major surgery in the UK Perioperative Quality Improvement Programme dataset^12^, which is a large dataset encompassing multiple procedure types and a general surgical population. Despite their model including variables derived from questionnaires which must be administered to patients by a clinician, our method’s performance exceeds their reported values.

Another large study of general surgical patients by Hur et al.^37^ used gradient boosting of decision trees^38^ to predict postoperative opioid use based on ICD and CPT codes in preoperative insurance claims data, with relatively modest predictive value. Nonetheless, their study suggests that there is value in using preoperative data to predict postoperative pain-related outcomes.

Modeling outcomes using CPT and ICD-10 codes requires an effective numerical representation. Studies by other groups on predicting postoperative outcomes using coding data from the EHR typically encode the presence or absence of a selected set of codes as binary variables^37,39–42^. However, this often necessitates identifying a priori a set of specific ICD-10 or CPT codes as candidate markers of risk, a process which we have found frequently overlooks codes with predictive value, or does not well align with actual coding practices found at a given institution. Without judicious selection of codes, this representation generates extremely high-dimensional sparse vector representations, which are poor inputs^43^ to both logistic regression and gradient boosting models^44^.

Our method utilizes the attention-based deep multiple instance learning framework of Ilse et al.^14^, and uses multi-head self-attention^15^ as a drop-in component which we select for its good empirical performance across a wide range of applications. Multiple instance learning is a paradigm which assigns labels to collections of datapoints rather than individual elements, where typically only a subset of those elements is informative. Variants of these methods are able to scale to sets of over a billion elements^45^, though in this application, there is only one procedure code and at most a few dozen comorbidities per patient. We model each patient’s collection of CPT and ICD-10 codes as an unordered set; without positional embeddings, multi-head attention learns a permutation-invariant set embedding which represents the information contained in each individual diagnosis or procedure as well as interactions between each pair of codes. Attention weights computed by the network can identify the most informative individual contributors in each patient’s set of codes.

Empirically, predictions made by POPS appear robust across a wide range of surgeries, patient populations, and between hospitals. We achieve this performance with a relatively simple network architecture with few enough parameters that it can be trained on a single GPU in a few minutes. Inference using a trained model requires negligible computational resources.

In the prospective cohort, the performance of our model in predicting postoperative pain levels was poorer than that achieved in the retrospective cohort on postoperative days 0 and 1. Model calibration and the distribution of outcomes indicates that there has been some drift in the distribution of postoperative pain outcomes between the retrospective and prospective cohorts; the prospective cohort who underwent surgery in 2023 had higher postoperative pain on average than those in the retrospective dataset between 2016–2020. A change over time in the composition of general surgical patients could be responsible for the differences in predictive performance of our model; there is a higher fraction of general surgery, neurosurgery, and thoracic surgery cases in the prospective cohort than in the retrospective dataset, which may represent on average more inherently painful cases. While we attempted to obtain a representative sample of surgical cases in our prospective study, it is also possible that some sampling bias was introduced in the process of enrolling patients. For example, two operating rooms at MGH with MRI machines could not be accessed for our prospective study, potentially skewing the distribution of cases. Finally, the fact that patients in the retrospective cohort received overall less intraoperative hydromorphone (Supplementary Fig. 8) may indicate that patients in the prospective cohort underwent more painful surgeries. Alternatively, this difference in intraoperative opioid administration may reflect a change in practice patterns that could also influence the postoperative pain trajectories of the prospective cohort^24^. Changes in practice likely occurred over the 3-year gap^46^; the COVID-19 pandemic resulted in fewer elective procedures performed and has had lasting effects on the distribution of surgical cases^47,48^. Therefore, periodic refitting may be necessary to maintain good performance and calibration. As the variables which POPS relies upon are routinely collected and can be extracted from the EHR without the need for manual chart review, this does not necessarily pose a difficult obstacle.

Predictions made by POPS outperformed those made by clinicians of the anesthesia team. The difference in performance increased after postoperative day 1, particularly for predictions of moderate and severe pain. One possible cause for this is that anesthesiologists typically do not receive information about patient recovery beyond the first postoperative day, especially for low-risk surgeries.

Another possibility is that postoperative days 0 and 1 are more influenced by intraoperative management than subsequent days^10^. For instance, a patient who receives intraoperative analgesics, or other interventions for pain management early in the postoperative recovery phase, may exhibit less pain than expected based on preoperative information. Once the effects of these interventions subside, the patient’s postoperative pain trajectory may regress to the mean. Consequently, if anesthesia care providers are making predictions that are primarily driven by their treatment plan, this would explain the drop in accuracy of clinician predictions past postoperative day 1, and the increase in model prediction performance.

There are inherent limitations to the degree to which postoperative pain can be predicted using only preoperative data. Intraoperative management of nociception may have causal effects on postoperative pain trajectories^49,50^, and we have shown in previous work that patterns of intraoperative opioid administration have changed over time^24^. Other factors such as surgical duration^51^, technique, or blood loss^52^ may also influence pain trajectories, and it is important to note that POPS predicts postoperative pain after standard-of-care treatment. Preoperative data alone is unable to account for variance in postoperative pain introduced by these variables. Moreover, pain is an inherently subjective phenomenon. The perception of pain varies on an individual basis in ways that cannot be fully accounted for and are not fully understood at present, and pain scales including the NRS are dependent upon patient self-report. Yet, POPS achieves reasonably good predictive performance on a difficult problem using only commonly available preoperative EHR data. Although our model outperformed clinicians’ predictions and reported performance metrics of other models in the literature, direct comparison against these methods on the same patients and outcomes was not possible with the available data. Our power calculations for required sample size are only a rough approximation. By using residual variance from the population mean, we don’t factor in clinicians’ ability to use preoperative information to predict postoperative pain. On the other hand, we also assume that clinicians have perfect knowledge of the population outcome distribution and are perfectly calibrated in their predictions. Depending on the clinical setting, these factors could lead to an underestimation or overestimation of required sample size. Nonetheless, we were able to find significant differences in performance in our prospective study. Because our model is not causal, learned associations between CPT or ICD-10 codes and postoperative pain outcomes may be confounded. Moreover, while attention weights broadly identify the most informative CPT or ICD-10 codes, due to collinearities between codes or low prevalence of specific combinations of codes, the estimated effect of a particular code may be uncertain. For example, though Patient 3’s history of depression has a significantly positive effect on expected pain on postoperative days 1–3, the associated ICD-10 code has a lower attention weight than other codes whose effect is not significant. Finally, although our model was developed using data from multiple centers, all study hospitals were from the greater Boston area, and therefore our study cohort may not be fully representative of the general surgical population in the United States. Coding practices specific to those institutions or the period over which data was collected may influence our results.

In conclusion, POPS provides a method for computational prediction of postoperative pain that could be applied in patients scheduled for a broad range of surgeries without increasing clinician burden and achieves state-of-the-art performance on a difficult prediction task. Our prospective study also shows the possible necessity of periodic model refitting after changes in patient population or practice patterns to maintain good performance and calibration. Overall, this work may aid in guiding interventions and further screening resources towards patients at high risk of high postoperative pain outcomes. In conjunction with our continuing work on characterizing the effects of intraoperative interventions on pain-related outcomes, we ultimately seek to further the development of objective pain management protocols to improve practice in perioperative pain management.

## Methods

A transparent reporting of a multivariable prediction model for individual prognosis or diagnosis (TRIPOD)^53^ checklist for this study is included in Supplementary Methods. There are two components to this study: a retrospective component with random split-sample development and validation, and a prospective component with validation only. The protocol for the retrospective component of this study and a waiver of informed consent for participants were approved by the Massachusetts General Hospital (MGH) institutional review board (IRB #2020P000301). Our prospective validation protocol was also approved by the MGH IRB (#2022P002958). Clinicians provided informed consent before participation in the prospective study. Study protocols are further detailed in Supplementary Methods. No deviations from these protocols occurred.

### Data extraction, processing, and study population

Our retrospective study included adult patients who underwent non-cardiac surgery with general anesthesia across two quaternary care academic medical centers, MGH and BWH, and two community hospitals, NSMC and NWH, between April 1^st^, 2016, and March 31^st^, 2020. We excluded patients admitted to the Intensive Care Unit immediately after the surgery and patients who died during the surgery.

Our prospective study included adult patients who underwent inpatient non-cardiac surgery with general anesthesia at MGH between February 15^th^, 2023, and March 20^th^, 2023. We excluded patients undergoing ambulatory surgery, non-elective surgery, or cardiac surgery. We also excluded patients admitted to the Intensive Care Unit immediately after the surgery and patients who died during the surgery.

Clinicians from the anesthesia team were surveyed at the beginning of each case, and their predictions on expected postoperative pain recorded (Supplementary Methods). We also recorded their role (attending, resident, or CRNA), years of experience (<5, 5–10, and >10 years), and gender. The research team did not intervene in the clinical management of these patients.

We employed data from patient electronic health records, and extracted demographics (age, weight, height, sex, race) and preoperative variables (preoperative pain score, surgery service, surgery urgency, and inpatient or ambulatory status). ICD-10 codes recorded prior to the date of surgery and CPT codes associated with each surgery were also extracted from the EHR, along with outcome data. EHR data for all patients was extracted from the Mass General Brigham (MGB) institutional Enterprise Data Warehouse (EDW) system and analytical platform.

From the set of CPT and ICD-10 codes present across all patient records in the retrospective dataset, a dictionary of all codes present in at least 1 in 10,000 records was built, comprised of 5,802 unique codes (999 CPT, 4,803 ICD-10). For each patient, their set of unique codes present both in the dictionary and their electronic health records was computed.

### Outcomes

The outcome studied were the maximal post-operative pain scores reported by patients on the day of the surgery (Postoperative Day 0), and on the four subsequent days (Postoperative Day 1 through 4). Pain is generally assessed using the Numeric Rating Scale (NRS)^54^, although in some cases is reported as strings. In these cases, we converted strings into numeric variables using 6 categories (“no pain” - 0, “mild pain” - 2, “moderate pain” - 4, “severe pain” - 6, “very severe pain” - 8, and “worst possible pain” - 10). If multiple pain scores were recorded on a given day for a single patient, the highest value was kept. Since pain scores were extracted directly from the EHR, no blinding was required.

### Network Structure

Set embeddings were computed using a neural network consisting of an embedding layer, which accepts as inputs the indexed set of CPT and ICD-10 codes of a given patient, and computes a 130 by 256 zero-padded matrix representation, where each row corresponds to the embedding of a specific CPT or ICD-10 code (Fig. 1). This is passed to a multi-head self-attention layer with mean pooling^15^. For each patient, this layer produces a single 256-dimensional vector which represents the information contained within their set of CPT and ICD-10 codes, which we refer to as the set embedding.

The set embedding is then concatenated with the normalized vector of patient demographic information and preoperative variables and passed to a densely connected feed-forward network. This feed-forward network predicts maximum postoperative pain score on the day of surgery (Postoperative Day 0), and on four subsequent days (Postoperative Days 1–4).

### Model Development

Patients in the retrospective dataset were sampled uniformly at random into training (81% of patients, *n* = 190,014), validation (9% of patients, *n* = 20,867), and test sets (10% of patients, *n* = 23,393). The validation set was used for model selection. Reported performance for the retrospective component of our study was evaluated on the test sets. The prospective cohort (*n* = 365) was only used for model evaluation. We also developed hospital-specific models, in which models were trained and selected using data from only a single hospital. We also evaluated performance on only the subset of the test set that was drawn from single hospitals.

Network parameters were learned through batch gradient descent with a batch size of 128. For binarized outcomes (moderate and severe pain, i.e. NRS > 4 and NRS > 6 respectively), our training objective function was binary cross-entropy. For continuous outcomes, our training objective function was mean squared error.

The training dataset was augmented by duplicating each patient without CPT codes. Without augmentation, the distribution of patients without CPT codes in the training set is limited to patients who underwent uncommon procedures. This impacts the computed impact of CPT codes in Table 3. The validation and test sets were not augmented.

### Hypothetical example patients

We defined three hypothetical example patients with demographic information, preoperative variables, and CPT and ICD codes required to compute the POPS (Table 3). Patient information was outlined by an anesthesiologist who aimed to characterize three archetypical subjects with different expected postoperative pain profiles based on existing clinical knowledge.

Attention weights for each patient’s CPT and ICD-10 codes were computed using our fitted model. The effect of each individual code was estimated for these patients by computing the difference in predicted outcomes for an identical patient with that code removed from their set.

### Statistical methods

Statistical significance of differences in area under receiver operating curves was assessed using Delong’s test^55^. Williams’ test was used to assess statistical significance of differences in Pearson’s correlation coefficients^56^. The Wilcoxon rank-sum test was used to assess significance of differences in root mean squared error. Differences in pain score distributions between the retrospective and prospective cohorts were assessed using the Chi-squared test. Differences in distributions of categorical variables between the retrospective and prospective cohorts were assessed using the Chi-squared test. Differences in distributions of normally distributed random variables (age, height, weight) were assessed using the t-test. Confidence bounds were computed by bootstrap.

Power calculations were performed using G*Power 3.1.9.7 to estimate the minimum number of patients required for our prospective study to identify significant differences in predictive performance between clinicians and our model. We computed the required sample size and parameters estimated from the empirical residual distribution of our model-based predictions of postoperative pain in the retrospective cohort. To estimate the residual distribution of clinician predictions, we computed the residual distribution for predicting the population mean pain for every patient. For a power of 0.90 and an α of 0.05 in a paired signed-rank test of performance between our model and clinician predictions, we estimated a required sample size of 365 patients.

### Reporting summary

Further information on research design is available in the Nature Research Reporting Summary linked to this article.

### Supplementary information

Supplemental Material

Reporting Summary

## Data Availability

Access to data used in this study requires a Data Use Agreement and IRB approval by the study institutions (MGB). Contingent upon these requirements, data are available from the authors upon reasonable request.

## Code availability

Code for algorithm development, evaluation, and statistical analysis will be made available without access restrictions at https://github.com/instigatorofawe/pain_prediction_pops after publication.
