# Supplementary material for: Development and prospective validation of postoperative pain prediction from preoperative EHR data using attention-based set embeddings
Source: NPJ Digit Med. 2023 Nov 16;6:209. doi: 10.1038/s41746-023-00947-z (PMC10654400; doi:10.1038/s41746-023-00947-z)
Supplement: Supplementary file 1 — Supplemental Material [file 41746_2023_947_MOESM1_ESM.docx]

**Development and validation of postoperative pain prediction from EHR data using attention-based set embeddings.**

Ran Liu^1,2†^, Rodrigo Gutiérrez^1,2†^, Rory V. Mather^1,2,3^, Tom A.D. Stone^1,2^, Laura A. Santa Cruz Mercado^1,2,4^, Kishore Bharadwaj^1,2^, Jasmine Johnson^1,2^, Proloy Das^1,2^, Gustavo Balanza^1,2^, Ekenedilichukwu Uwanaka^1,2^, Justin Sydloski^1,2^, Andrew Chen^1,2^, Mackenzie Hagood^1,2^, Edward A. Bittner^1,2^, and Patrick L. Purdon^1,2*^.

**Supplementary Materials**

**Table of Contents**

[Supplementary Methods 4](#_Toc146623531)

[Clinician survey 4](#_Toc146623532)

[Retrospective Protocol 6](#_Toc146623533)

[Prospective Protocol 11](#_Toc146623534)

[TRIPOD Checklist for Prediction Model Development 14](#_Toc146623535)

[Supplementary Figures 16](#_Toc146623536)

[Supplementary Figure 1. Distribution of pain scores by type in the retrospective (A) and prospective (B) cohorts. Cyan represents pain string and red represents pain as number. 16](#_Toc146623537)

[Supplementary Figure 2. Distribution of pain score outcomes. Red represents the prospective cohort and cyan represents the retrospective cohort. 17](#_Toc146623538)

[Supplementary Figure 3. Number of pain score observations per patient on each postoperative day for patients with at least 1 pain score observation. Red represents the prospective cohort, green represents the inpatient retrospective cohort and blue represents the outpatient retrospective cohort. 18](#_Toc146623539)

[Supplementary Figure 8. Total intraoperative dosage of fentanyl and hydromorphone for surgeries at MGH in the retrospective and prospective cohorts. Red represents the prospective cohort and cyan represents the retrospective cohort. 23](#_Toc146623540)

[Supplementary Tables 24](#_Toc146623541)

[Supplementary Table 1. Number of patients with outcome data, and number of patients excluded on each postoperative day. 24](#_Toc146623542)

[Supplementary Table 2. Most frequent CPT codes in the retrospective study cohort. 25](#_Toc146623543)

[Supplementary Table 3. Most frequent ICD-10 codes in the retrospective cohort. 26](#_Toc146623544)

[Supplementary Table 4. Most frequent CPT codes in the prospective cohort. 27](#_Toc146623545)

[Supplementary Table 5. Most frequent ICD-10 codes in the prospective cohort. 28](#_Toc146623546)

[Supplementary Table 6. Baseline statistics for the subgroup of patients with outcomes on postoperative day 0. MGH: Massachusetts General Hospital, BWH: Brigham and Women’s Hospital, NSMC: North Shore Medical Center, NWH: Newton Wellesley Hospital, ASA: American Society of Anesthesiologist Performance Status. 29](#_Toc146623547)

[Supplementary Table 7. Baseline statistics for the subgroup of patients with outcomes on postoperative day 1. MGH: Massachusetts General Hospital, BWH: Brigham and Women’s Hospital, NSMC: North Shore Medical Center, NWH: Newton Wellesley Hospital, ASA: American Society of Anesthesiologist Performance Status. 31](#_Toc146623548)

[Supplementary Table 8. Baseline statistics for the subgroup of patients with outcomes on postoperative day 2. MGH: Massachusetts General Hospital, BWH: Brigham and Women’s Hospital, NSMC: North Shore Medical Center, NWH: Newton Wellesley Hospital, ASA: American Society of Anesthesiologist Performance Status. 33](#_Toc146623549)

[Supplementary Table 9. Baseline statistics for the subgroup of patients with outcomes on postoperative day 3. MGH: Massachusetts General Hospital, BWH: Brigham and Women’s Hospital, NSMC: North Shore Medical Center, NWH: Newton Wellesley Hospital, ASA: American Society of Anesthesiologist Performance Status. 35](#_Toc146623550)

[Supplementary Table 10. Baseline statistics for the subgroup of patients with outcomes on postoperative day 4. MGH: Massachusetts General Hospital, BWH: Brigham and Women’s Hospital, NSMC: North Shore Medical Center, NWH: Newton Wellesley Hospital, ASA: American Society of Anesthesiologist Performance Status. 37](#_Toc146623551)

[Supplementary Table 11. AUC by surgical service for moderate pain (max pain >4) in the retrospective cohort 39](#_Toc146623552)

[Supplementary Table 12. AUC by surgical service for severe pain (max pain >6) in the retrospective cohort 40](#_Toc146623553)

[Supplementary Table 13. RMSE by surgical service in the retrospective cohort 41](#_Toc146623554)

[Supplementary Table 14. Correlation by surgical service in the retrospective cohort 42](#_Toc146623555)

[Supplementary Table 15. AUC in the retrospective cohort for moderate pain (max pain >4). MGH: Massachusetts General Hospital, BWH: Brigham and Women’s Hospital, NSMC: North Shore Medical Center, NWH: Newton Wellesley Hospital. 43](#_Toc146623556)

[Supplementary Table 16. AUC in the retrospective cohort for severe pain (max pain >6). MGH: Massachusetts General Hospital, BWH: Brigham and Women’s Hospital, NSMC: North Shore Medical Center, NWH: Newton Wellesley Hospital. 43](#_Toc146623557)

[Supplementary Table 17. RMSE in the retrospective cohort. MGH: Massachusetts General Hospital, BWH: Brigham and Women’s Hospital, NSMC: North Shore Medical Center, NWH: Newton Wellesley Hospital. 43](#_Toc146623558)

[Supplementary Table 18. Correlation in the retrospective cohort. MGH: Massachusetts General Hospital, BWH: Brigham and Women’s Hospital, NSMC: North Shore Medical Center, NWH: Newton Wellesley Hospital. 44](#_Toc146623559)

[Supplementary Table 19. O/E ratio for moderate pain (max pain >4) with 95% confidence intervals. 44](#_Toc146623560)

[Supplementary Table 20. O/E ratio for severe pain (max pain >6) with 95% confidence intervals. 44](#_Toc146623561)

[Supplementary Table 21. Calibration intercept, with 95% confidence intervals. 45](#_Toc146623562)

[Supplementary Table 22. Calibration slope, with 95% confidence intervals 45](#_Toc146623563)

[Supplementary Table 23. AUC in the prospective cohort for moderate pain (max pain >4). MGH: Massachusetts General Hospital, BWH: Brigham and Women’s Hospital, NSMC: North Shore Medical Center, NWH: Newton Wellesley Hospital. 46](#_Toc146623564)

[Supplementary Table 24. AUC in the prospective cohort for severe pain (max pain >6). MGH: Massachusetts General Hospital, BWH: Brigham and Women’s Hospital, NSMC: North Shore Medical Center, NWH: Newton Wellesley Hospital. 46](#_Toc146623565)

[Supplementary Table 25. RMSE in the prospective cohort. MGH: Massachusetts General Hospital, BWH: Brigham and Women’s Hospital, NSMC: North Shore Medical Center, NWH: Newton Wellesley Hospital. 46](#_Toc146623566)

[Supplementary Table 26. Correlation in the prospective cohort. MGH: Massachusetts General Hospital, BWH: Brigham and Women’s Hospital, NSMC: North Shore Medical Center, NWH: Newton Wellesley Hospital. 47](#_Toc146623567)

# Supplementary Methods

## Clinician survey

The following questionnaire is part of a study that aims to validate a prediction model to forecast postoperative pain. Your participation by answering this survey is completely **voluntary** and **anonymous**. All information is de-identified and securely stored according to MGH policies. This study was reviewed and approved by the MGH IRB.

Voluntarily continuing with this questionnaire means that you are giving your consent to participate in the present study. However, you can withdraw at any time from the study by simply skipping the survey.

The questionnaire will take around 3 minutes to answer.

---o---

Gender of anesthesia provider:

*(Mark one option)*

- Female
- Male
- Other

How many years of experience do you have providing anesthetic care (including your training years)?

*(Mark one option)*

- Less than 5
- Between 5 and 10 years
- Between 10 and 20 years
- More than 20 years

On a scale from 0 to 10, with 0 no pain and 10 the most intense pain imaginable, regarding the patient that is now in the OR under your care:

Based on your experience and the patient’s characteristics, which is the **maximal pain score** you predict that this patient will report on **the day of surgery**?

*(Mark one option)*

0 1 2 3 4 5 6 7 8 9 10

Which is the **maximal pain score** you predict that this patient will report **during PACU stay**?

*(Mark one option)*

0 1 2 3 4 5 6 7 8 9 10

Which is the **maximal pain score** you predict that this patient will report on **postoperative day 1**?

*(Mark one option)*

0 1 2 3 4 5 6 7 8 9 10

Which is the **maximal pain score you** predict that this patient will report on **postoperative day 2**?

*(Mark one option)*

0 1 2 3 4 5 6 7 8 9 10

Which is the **maximal pain score** you predict that this patient will report on **postoperative day 3**?

*(Mark one option)*

0 1 2 3 4 5 6 7 8 9 10

Which is the maximal pain score you predict that this patient will report on **postoperative day 4**?

*(Mark one option)*

0 1 2 3 4 5 6 7 8 9 10

## Retrospective Protocol

The retrospective part of the current study is a secondary analysis of the protocol “A Retrospective Cohort Study of Intra-operative Opioid Administration and Pain Related Outcomes in the Post Anesthesia Care Unit” which is already published (Santa Cruz Mercado et al. JAMA 2023), including its Statistical Analysis Plan.

The specific considerations for the current study are the following:

**Study design:** Retrospective Cohort Study.

**Locations:**

- Massachusetts General Hospital
- Brigham and Women’s Hospital
- North Shore Medical Center
- Newton Wellesley Hospital

**Subjects:** All adult patients undergoing non-cardiac surgery under general anesthesia between April 1^st^, 2016, and March 31^st^, 2020.

**Inclusion Criteria:** Adult patients undergoing non-cardiac surgery under general anesthesia.

**Exclusion Criteria:** Patients admitted to the ICU after the surgery, patients who died during the surgery.

**Variable definition:**

| **Group** | **Variable Name** | **Definition** | **Extraction Summary** | **Variable Type** |
| --- | --- | --- | --- | --- |
| **POC.1 Outcomes** | | | | |
| **POC.1** | **Max Pain Score Day 0** | The maximum pain score within the day of the surgery.  See Time Windows Definitions. | Pain score records on day of surgery using the Numeric Rating Scale (values from 0 to 10). Sometimes clinicians recorded 0 pain as “no pain” string variable. | Categorical |
| **POC.2** | **Max Pain Score Day 1** | The maximum pain score within the first day after the surgery.  See Time Windows Definitions. |  |  |
| **POC.3** | **Max Pain Score Day 2** | The maximum pain score within the second day after the surgery.  See Time Windows Definitions. |  |  |
| **POC.4** | **Max Pain Score Day 3** | The maximum pain score within the third day after the surgery.  See Time Windows Definitions. |  |  |
| **POC.5** | **Max Pain Score Day 4** | The maximum pain score within the fourth day after the surgery.  See Time Windows Definitions. |  |  |
| **PTBV.1 Demographics** | | | | |
| **PTBV.1** | **Age** | The age of the patient on the day of surgery. | Date of service timestamp - date of birth timestamp | Numeric |
| **PTBV.1** | **Gender** | The gender (male or female) of the patient on the day of the surgery. | Gender record | Binary |
| **PTBV.1** | **Race** | The race of the patient on the day of surgery | Race record | Categorical |
| **PTBV.1** | **Height** | The height of the patient on the day of surgery (in inches). If the height is unavailable on the day of surgery, the height last recorded was obtained. | Height records | Numeric |
| **PTBV.1** | **Weight** | The weight of the patient on the day of surgery (in kg). If the weight is unavailable on the day of surgery, the weight last recorded was obtained. | Height and weight records | Numeric |
| **PTBV.2 Baseline Clinical Measures** | | | | |
| **PTBV.2** | **Pain Score at Last Visit** | A patient’s pain score (0-10) recorded in their most recent clinical visit prior to surgery. Patients without a recorded pain score prior to surgery are binned separately. | Pain score records prior to day of surgery | Categorical |
| **SBV Surgical Baseline Variables** | | | | |
| **SBV** | **Surgery Urgency** | Whether a particular surgery is defined as emergent, urgent, non-urgent, or elective. If patient records have the surgery urgency recorded, the record is used. Otherwise, the scheduled start time is compared to the actual procedure start time. Within 1 hr: Emergent, within 4 hrs: Urgent, within 24 hrs: non-urgent, otherwise elective. | Surgery Urgency record, procedure start timestamp, scheduled start timestamp | Categorical |
| **SBV** | **Inpatient Vs Ambulatory** | Whether a patient is classified as inpatient (1) or ambulatory (0). | Record for inpatient or ambulatory | Binary |
| **SBV** | **CPT Code** | The CPT code(s) associated with the procedure. | CPT records for the surgeries | Categorical Vector |
| **SBV** | **Surgery Service** | The surgical service (out of 24 possible services) that the particular surgery was classified under. | String Label | Categorical |
| **TIME Time-Stamps** | | | | |
| **TIME** | **Preop Start** | See Time Windows Definitions. | Hospital Admission | DateTime |
| **TIME** | **Preop End** |  | AnesthesiaStartDTS, AnesthesiaStart, or Start Data Collection | DateTime |
| **TIME** | **Preop Time End** |  | Patient In Room | DateTime |
| **TIME** | **Surgical Duration Start** |  | ProcedureStartDTS | DateTime |
| **TIME** | **Surgical Duration End** |  | ProcedureCompletedDTS | DateTime |
| **TIME** | **PACU Hold Start** |  | Extubation or Stop of CO2 Ventilator | DateTime |
| **TIME** | **PACU Hold End** |  | InPhase1 or In PACU | DateTime |
| **TIME** | **PACU Start** |  | InPhase1 or In PACU | DateTime |
| **TIME** | **PACU End** |  | Ready for PACU Discharge | DateTime |
| **TIME** | **In Hospital Start** |  | Hospital Admission | DateTime |
| **TIME** | **In Hospital End** |  | Hospital Discharge | DateTime |

**Time Windows definitions**

Multiple variables are considered within the context of certain time windows and events. Below are descriptions of time windows and events, as well an explanation of the programming logic used with collected EDW variables.

- 1. **Hospital Length of Stay:** The period from admission to discharge.

Start: HospitalAdmissionDTS

End: HospitalDischargeDTS

- 1. **Pre-Operative:** Occurring prior to the start of surgery but on the day of surgery.

Start: HospitalAdmissionDTS, CPC/PACU admission

End: AnesthesiaStartDTS, AnesthesiaStart, or Start Data Collection

- 1. **Surgical Duration**: The period of active surgery.

Start: ProcedureStartDTS

End: ProcedureCompletedDTS

- 1. **Intra-operative Anesthesia Care:** The intra-operative period during which opioid drugs are given to treat intra-operative nociception and as prophylaxis for postoperative pain. This period will be used as the *"Primary Exposure Window"*.

Start: ProcedureStartDTS (T_1_ in Figure 3 of the SAP)

End: Extubation or Stop of CO2 Ventilator + 10 minutes

- 1. **PACU Hold:** In the event that patients have completed their surgery but are unable to be transferred to the PACU, it is possible that post-operative treatment can begin while still in the operating room. To consider this, the PACU Hold window is defined.

Start: Extubation or Stop of CO2 Ventilator

End: InPhase1 or In PACU

- 1. **PACU Stay:** This is the period which patients are receiving initial post-operative care. This is also the time window in which we evaluated our primary outcomes.

Start: InPhase1 or In PACU

End: Ready for PACU Discharge (see below)

- 1. **PACU Stay for primary outcomes:** This is the period which patients are receiving initial post-operative care.

Start: Extubation or Stop of CO2 Ventilator + 10 minutes

End: Ready for PACU Discharge (see below)

- 1. **Ready For PACU Discharge:** The end of the PACU Stay time window. Ambulatory and Inpatient cases have different considerations for what denotes the end of their PACU stay. This is defined as follows:

Ambulatory:

- If only 1 Anesthesia Postprocedure Evaluation (APE) Note exists that is associated with a post-operative provider and the timestamp of the note is at least 1 hour from the start of PACU, we used this timestamp.
- If only 1 APE Note exists that is associated with the post-operative provider and the timestamp of the note is less than 1 hour from the start of PACU, we calculated the duration from the start of the PACU stay to the Phase II Care Complete and APE Note timestamp, and we chose the larger duration’s timestamp.
- If multiple APE Notes exist, we ignored the first note. Then we used the timestamp of the first note that is associated with a post-operative provider.
- If there are no APE Notes available on record or none of the above criteria apply, we used Phase II Care Complete.

Inpatient:

- If only 1 APE Note exists that is associated with a post-operative provider and the timestamp of the note is at least 1 hour from the start of PACU, we used this timestamp.
- If only 1 APE Note exists that is associated with the post-operative provider and the timestamp of the note is less than 1 hour from the start of PACU, we calculated the duration from the start of the PACU stay to the Phase I Care Complete and APE Note timestamp and chose the larger duration’s timestamp.
- If multiple APE Notes exist, we ignored the first note. Then we used the timestamp of the first note that is associated with a post-operative provider.
- If there are no APE Notes available on record or none of the above criteria apply, we used Phase I Care Complete.
  1. **Postoperative Day 0**: Corresponds to the period of time between the day of the surgery and the 11:59 pm of that same day.

Start: ProcedureCompletedDTS

End: 11:59 pm of that day

- 1. **Postoperative Day 1**: This applies only for inpatients and corresponds to the period of time between the day after the end of surgery and the following 24 hours.

Start: Day after ProcedureCompletedDTS

End: Start + 24 hours

- 1. **Postoperative Day 2**: This applies only for inpatients and corresponds to the period of time between the day after Postoperative Day 1 and the following 24 hours.

Start: Day after Postoperative Day 1

End: Start + 24 hours

- 1. **Postoperative Day 3**: This applies only for inpatients and corresponds to the period of time between the day after Postoperative Day 2 and the following 24 hours.

Start: Day after Postoperative Day 2

End: Start + 24 hours

- 1. **Postoperative Day 4**: This applies only for inpatients and corresponds to the period of time between the day after Postoperative Day 3 and the following 24 hours.

Start: Day after Postoperative Day 3

End: Start + 24 hours

**Sample size:** No sample size calculation was made. We included all patients meeting our inclusion criteria.

**Data extraction:** Medical records were extracted from the EPIC database. All data variables from the database were properly identified with a database atlas that included variable names and definitions and queried using SQL. Due to implementation of EPIC at MGH in 2016, we did not have access to data preceding this timepoint. Thus, we chose to begin our patient selection search criteria with timepoints beginning after April 2016.

We accessed the data using the Server Query Language (SQL) through the program Microsoft SQL Server Management Studio. As the scope of the study was quite broad, we queried for basic identifiers for all patients meeting the study’s inclusion criteria. The inclusion criteria of patients aged 18 years or older, date of surgery within April 2016 to March 2020, non-cardiac surgeries/procedures, and transferal to ICU were direct filters to implement in the query. Due to the mislabeling of non-general anesthesia cases, we created an algorithm to identify true general anesthesia cases within the database from mislabeled cases. We defined general anesthesia as the presence of any one of the following criteria between the ‘In Room’ and ‘Out of Room’ time points within the general anesthesia record in EPIC: Intubation Time Point, Extubation Time Point, LMA placement/removal or ETT tube placement and removal. We also included cases that were flagged as having an intubation. After removing cases that did not meet these criteria, we were left with 234274 surgical cases who went to the PACU or CPC after surgery. From this patient list, we then extracted all associated patient data and applied the remaining exclusion criteria in our MATLAB data processing pipeline.

## Prospective Protocol

1. **Background and Significance**

Of the 51 million patients who undergo surgery each year in the United States,^1^ as many as 80% experience acute postoperative pain,^2,3^ and a majority report inadequate pain relief_._^3^ Uncontrolled pain hinders postsurgical recovery, prolonging hospital stays, and increases mortality and the likelihood of chronic pain.^4,5^ On the other hand, acute pain is commonly managed with opioids, prescribed to over 80% of surgical patients;^1^ the risk of opioid use disorder, a present public health crisis,^6,7^ increases with dosage and duration.^1^ The American Pain Society recommends that clinicians individualize courses of treatment for each patient, yet existing assessments are heavily subjective, and many recommendations lack strong evidence.^8^ Computational prediction of postoperative pain can provide quantitative guidance for perioperative pain management, and focus interventions on cases at greatest risk of acute pain.

Existing literature on predicting postoperative pain is sparse and limited in scope. Only a handful of studies have attempted to compare pain across different procedure types^9^. Previous studies have used logistic regression to predict the likelihood of uncontrolled pain in relatively small cohorts of ambulatory^10^ and elective^11^ surgical cases, achieving moderate levels of performance. However, a major limitation of these studies is that they rely upon physician evaluations and patient surveys of anticipated pain, and thus require human input, and reflect the results of human prediction of postoperative pain more so than computational prediction.

In this study, we aim to compare the performance of a machine learning method for predicting postoperative pain in a wide range of inpatient surgeries using information about patients and procedures from commonly recorded preoperative electronic health record data with the predictions made by members of the anesthesia team.

1. **Specific Aims and Objectives**

**Aim 1.** To evaluate the accuracy of the prediction made by members of the anesthesia team to forecast postoperative pain of patients that they are overseeing in the Operating Room.

**Aim 2.** To compare the prediction performance of clinicians with a machine learning-based algorithm.

We hypothesized that a machine learning-based algorithm designed to predict postoperative pain will performance better than clinicians’ prediction.

1. **General Description of Study Design**

**Study design:** Prospective Cohort Study.

**Location:** Massachusetts General Hospital

1. **Subject Selection**

**Subjects:** Attendings, residents, and CRNAs in the Department of Anesthesia, Critical Care and Pain Medicine (DACCPM) at Massachusetts General Hospital (MGH).

**Inclusion/Exclusion Criteria:** Only attendings, residents, and CRNAs in the DACCPM at MGH are eligible to participate in this study.

**Subject Recruitment Procedures:** The subjects will be recruited from the DACCPM at MGH. Subjects will be informed by emails and/or in person discussions with the research team. We will make sure that all subjects understand that participation in this study is entirely voluntary. According to our sample size and considering the number of clinical research coordinators in our team, we expected to complete the enrollment of subjects in a 2-weeks period. All subjects will be recruit in MGH Main Campus facilities (OR area).

1. **Subject Enrollment**

**Consent:** Due to the minimal risk nature of this study, we have asked for a waiver of written consent. The risks and benefits for participating in the study are minimal. There are no immediate, delayed, or long-term physical, economic or psychological risks associated with completing the questionnaires that make up this voluntary study. Subjects **will be informed that their participation is entirely voluntary during recruitment** and provided with a fact sheet outlining the study. Furthermore, language highlighting the voluntary nature of the questionnaire is provided at the very start of each questionnaire. Subjects who continue with completing the questionnaire are assumed to have implied consent. Participants can withdraw at any time or contact the principal investigator for help/concerns or to ask for their responses to be excluded from the study.

1. **Study procedures**

Subject participation is carried out online via the completion of one survey. The survey will be administered securely through RedCap. The survey will ask the following questions:

- Position: Attending, Resident, CRNAs
- Years of experience providing anesthesia care
- Regarding the patient under your current care, what would you expect to be the maximal pain score reported in a scale from 0 to 10 on:
- The day of the surgery
- Postoperative day 1
- Postoperative day 2
- Postoperative day 3
- Postoperative day 4

No drugs, medical devices, procedure/surgical interventions will be used as part of this study.

**Outcomes:** The primary outcome will be the highest postoperative pain reported by the patient the day of the surgery. Secondary outcomes will include the highest postoperative pain reported by the patient on postoperative day 1, 2, 3 and 4. Pain score is usually evaluated with the Numeric Pain Rating Scale (NPRS), which is a unidimensional measure of pain, that goes from 0 (no pain) to 10 (worst pain imaginable). We will categorize NPRS as a binary outcome: below 4 and equal or higher than 4.

**Data collection:** The questionnaires responses will be automatically store in the RedCap database designed for this study. To assess the outcomes, we will obtain the deidentify information from patient electronic health records through systematic data queries using the MGH Enterprise Data Warehouse (EDW) system. To feed our machine learning model, we will also obtain deidentify data from patient electronic health records: CPT and ICD-10 codes, demographic, and preoperative data,

1. **Statistical Analysis**

**Statistical Methods:** We will compute the Area Under the Curve (AUC) of the Receiving Operators Curves (ROC) for both, clinicians’ predictions and our model, to predict moderate-severe pain (i.e., NPRS > 3). Sensibility and Specificity will be also estimated for both methods.

**Power Analysis:** We computed required sample size using G*Power 3.1.9.7, and parameters estimated from the empirical residual distribution of our model-based predictions of postoperative pain. To estimate the residual distribution of clinician predictions, we computed the residual distribution for predicting the population mean pain for every patient. For a power of 0.90 and an α of 0.05 in a paired signed-rank test of performance between our model and clinician predictions, we estimate a required sample size of 365 patients.

1. **Monitoring and Quality Assurance**

Monitoring the validity and integrity of the data and adherence to the IRB-approved protocol will be the primary responsibility of the Principal Investigator, Patrick L. Purdon, PhD. The Principal Investigator will guarantee strict adherence to the IRB-approved protocol and will monitor the integrity of the data collected. The Principal Investigator will assess the quality and completeness of aggregate questionnaire data regularly over the course of the study. Potential problems will be identified and corrected as needed.

The confidentiality of subject data will be discussed actively and longitudinally as needed during the period that the study is active. There are no immediate, delayed, or long-term physical, economic or psychological safety issues associated with this study. The probability and magnitude of harm or discomfort anticipated in completing the questionnaires is minimal. Should an adverse event or other unanticipated problem need to be reported, the Principal Investigator will be responsible for reporting in a timely manner according to the IRB regulations. All adverse events will be reported through appropriate channels of the MGH Human Research Committee. Unanticipated problems involving risks to subjects or others including adverse events will be reported to the PHRC as described in the PHRC policy on Adverse Event Reporting and Unanticipated Problems Involving Risks to Subjects or Others.

## TRIPOD Checklist for Prediction Model Development

| **Section/Topic** | **Item** | **Checklist Item** | **Page** |
| --- | --- | --- | --- |
| **Title and abstract** | | | |
| Title | 1 | Identify the study as developing and/or validating a multivariable prediction model, the target population, and the outcome to be predicted. | 1 |
| Abstract | 2 | Provide a summary of objectives, study design, setting, participants, sample size, predictors, outcome, statistical analysis, results, and conclusions. | 2 |
| **Introduction** | | | |
| Background and objectives | 3a | Explain the medical context (including whether diagnostic or prognostic) and rationale for developing or validating the multivariable prediction model, including references to existing models. | 3 |
|  | 3b | Specify the objectives, including whether the study describes the development or validation of the model or both. | 3 |
| **Methods** | | | |
| Source of data | 4a | Describe the study design or source of data (e.g., randomized trial, cohort, or registry data), separately for the development and validation data sets, if applicable. | 11 |
|  | 4b | Specify the key study dates, including start of accrual; end of accrual; and, if applicable, end of follow-up. | 11 |
| Participants | 5a | Specify key elements of the study setting (e.g., primary care, secondary care, general population) including number and location of centres. | 11 |
|  | 5b | Describe eligibility criteria for participants. | 11 |
|  | 5c | Give details of treatments received, if relevant. | 11 |
| Outcome | 6a | Clearly define the outcome that is predicted by the prediction model, including how and when assessed. | 12 |
|  | 6b | Report any actions to blind assessment of the outcome to be predicted. | 12 |
| Predictors | 7a | Clearly define all predictors used in developing or validating the multivariable prediction model, including how and when they were measured. | 12 |
|  | 7b | Report any actions to blind assessment of predictors for the outcome and other predictors. | NA |
| Sample size | 8 | Explain how the study size was arrived at. | 13 |
| Missing data | 9 | Describe how missing data were handled (e.g., complete-case analysis, single imputation, multiple imputation) with details of any imputation method. | 12 |
| Statistical analysis methods | 10a | Describe how predictors were handled in the analyses. | 12 |
|  | 10b | Specify type of model, all model-building procedures (including any predictor selection), and method for internal validation. | 12-13 |
|  | 10d | Specify all measures used to assess model performance and, if relevant, to compare multiple models. | 13-14 |
| Risk groups | 11 | Provide details on how risk groups were created, if done. | NA |
| **Results** | | | |
| Participants | 13a | Describe the flow of participants through the study, including the number of participants with and without the outcome and, if applicable, a summary of the follow-up time. A diagram may be helpful. | Suppl. Table 1 |
|  | 13b | Describe the characteristics of the participants (basic demographics, clinical features, available predictors), including the number of participants with missing data for predictors and outcome. | 4, Table 1 |
| Model development | 14a | Specify the number of participants and outcome events in each analysis. | 4 |
|  | 14b | If done, report the unadjusted association between each candidate predictor and outcome. | NA |
| Model specification | 15a | Present the full prediction model to allow predictions for individuals (i.e., all regression coefficients, and model intercept or baseline survival at a given time point). | NA |
|  | 15b | Explain how to the use the prediction model. | 12-13 |
| Model performance | 16 | Report performance measures (with CIs) for the prediction model. | 5-6, Figures 2-3 |
| **Discussion** | | | |
| Limitations | 18 | Discuss any limitations of the study (such as nonrepresentative sample, few events per predictor, missing data). | 10 |
| Interpretation | 19b | Give an overall interpretation of the results, considering objectives, limitations, and results from similar studies, and other relevant evidence. | 7-10 |
| Implications | 20 | Discuss the potential clinical use of the model and implications for future research. | 7-8 |
| **Other information** | | | |
| Supplementary information | 21 | Provide information about the availability of supplementary resources, such as study protocol, Web calculator, and data sets. | 14 |
| Funding | 22 | Give the source of funding and the role of the funders for the present study. | 15 |

# Supplementary Figures

# Supplementary Figure 1. Distribution of pain scores by type in the retrospective (A) and prospective (B) cohorts. Cyan represents pain string and red represents pain as number.


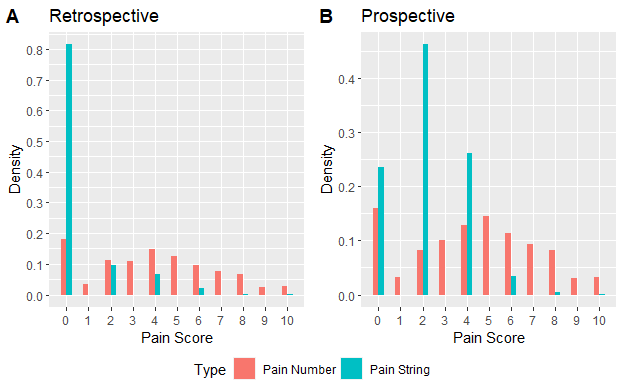


# Supplementary Figure 2. Distribution of pain score outcomes. Red represents the prospective cohort and cyan represents the retrospective cohort.


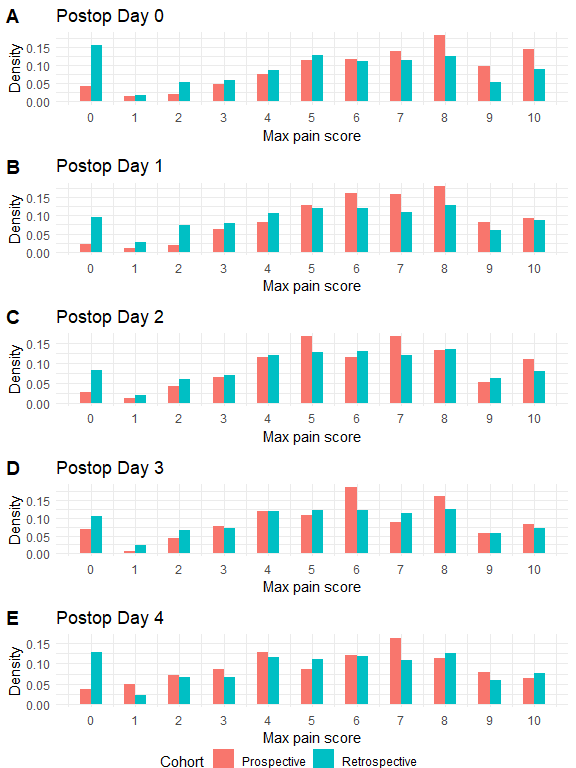


# Supplementary Figure 3. Number of pain score observations per patient on each postoperative day for patients with at least 1 pain score observation. Red represents the prospective cohort, green represents the inpatient retrospective cohort and blue represents the outpatient retrospective cohort.


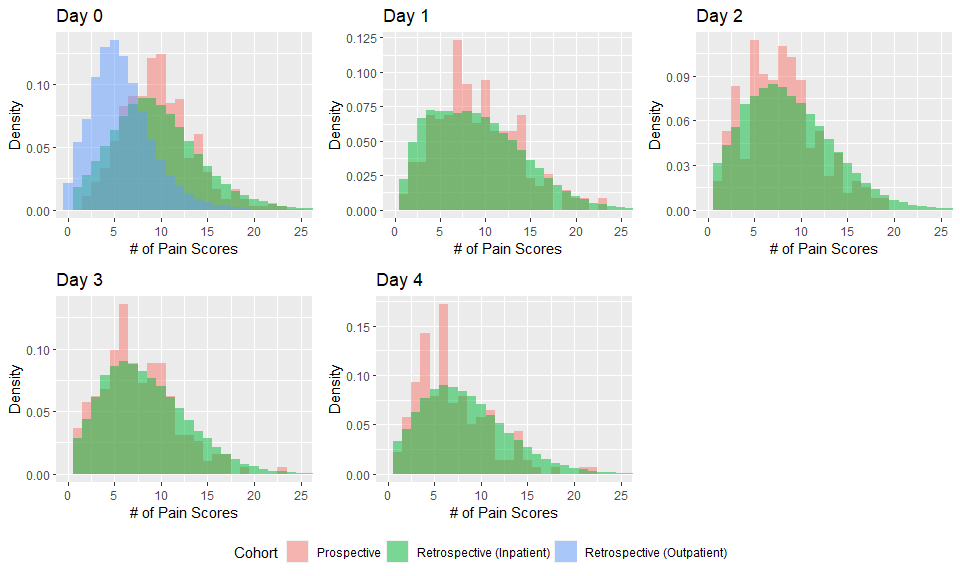


**Supplementary** Figure 4. Distribution of outcomes on each postoperative day for patients whose last observation of pain score occurred on each of the 5 postoperative days. Lines and dots indicate mean values on each day. Vertical bars and shaded areas indicate an interval of 1 standard deviation. Each color represents different postoperative day.


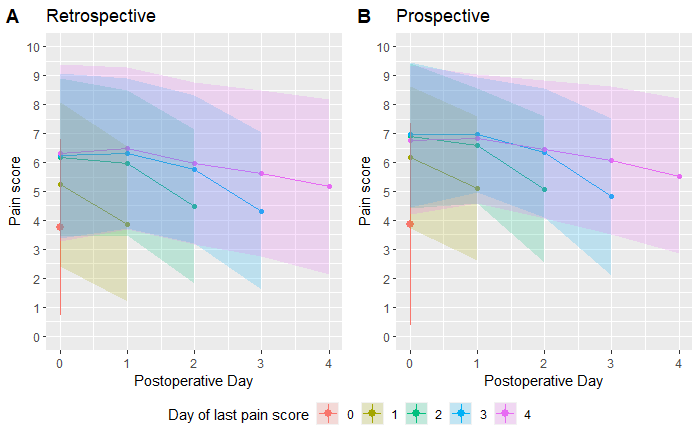


**Supplementary** Figure 5. Calibration plots for postoperative pain predictions in the retrospective cohort. Moving averages are indicated by the blue line. The red line indicates perfect calibration, with a slope of 1 and intercept of 0.


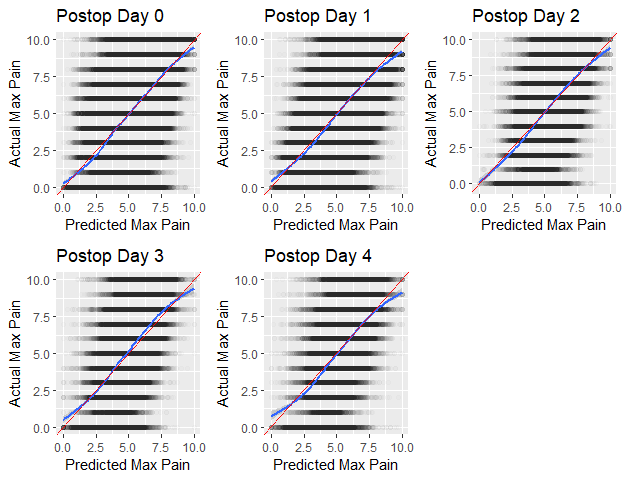


**Supplementary** Figure 6. Calibration plots for postoperative pain predictions in the prospective cohort. Moving averages are indicated by the blue line. The red line indicates perfect calibration, with a slope of 1 and intercept of 0.


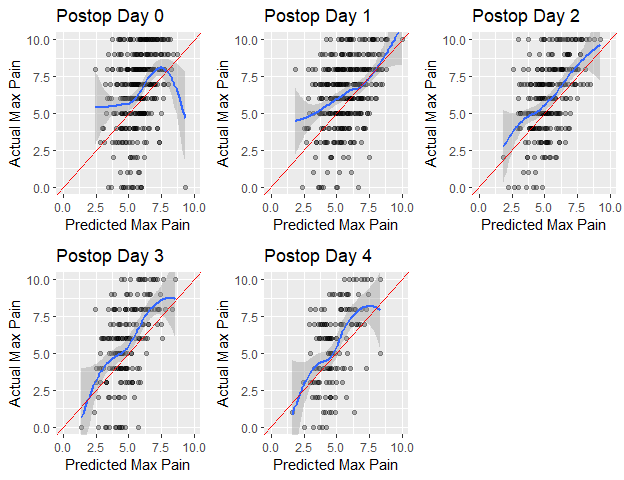


**Supplementary** Figure 7. Calibration plots for clinician predictions of postoperative pain in the prospective cohort. Moving averages are indicated by the blue line. The red line indicates perfect calibration, with a slope of 1 and intercept of 0.


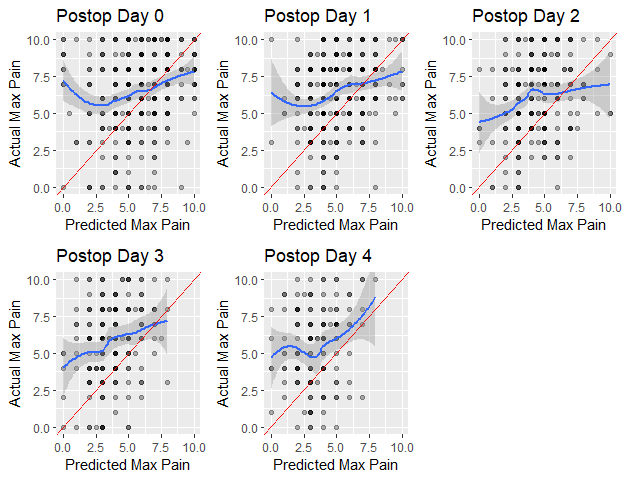


# Supplementary Figure 8. Total intraoperative dosage of fentanyl and hydromorphone for surgeries at MGH in the retrospective and prospective cohorts. Red represents the prospective cohort and cyan represents the retrospective cohort.


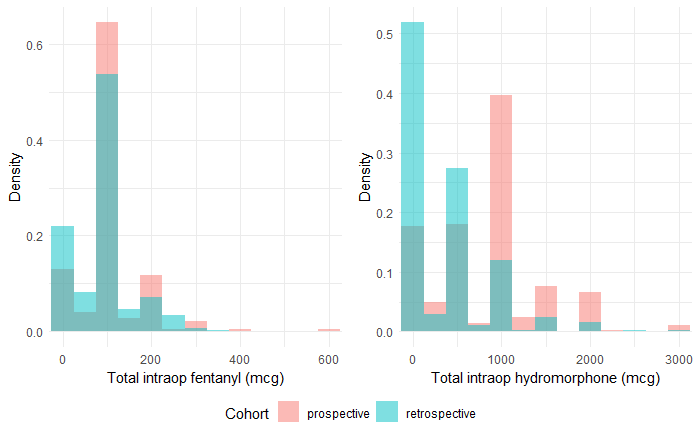


# Supplementary Tables

# Supplementary Table 1. Number of patients with outcome data, and number of patients excluded on each postoperative day.

| **Postop Day** | **Retrospective** | **Pain >4** | **Pain >6** | **Excluded** | **Prospective** | **Pain >4** | **Pain >6** | **Excluded** |
| --- | --- | --- | --- | --- | --- | --- | --- | --- |
| 0 | 217,096 | 135,762 | 83,195 | 28,762 | 344 | 274 | 195 | 22 |
| 1 | 158,200 | 98,663 | 60,933 | 87,658 | 350 | 280 | 179 | 16 |
| 2 | 96,364 | 62,799 | 37,926 | 149,494 | 264 | 196 | 122 | 102 |
| 3 | 70,216 | 42,991 | 25,761 | 175,642 | 192 | 132 | 75 | 174 |
| 4 | 50,326 | 30,232 | 18,644 | 195,532 | 140 | 88 | 59 | 226 |

# Supplementary Table 2. Most frequent CPT codes in the retrospective study cohort.

| CPT | Description | n | Frequency (%) |
| --- | --- | --- | --- |
| 31622 | Brnchsc incl fluor gdnce dx w/cell washg spx | 8,210 | 3.34 |
| 47562 | Llaparoscopic, cholecystectomy | 6,253 | 2.54 |
| 58661 | Llaparoscopic, rmv adnexal structure | 4,669 | 1.90 |
| 29881 | Knee scope, med or lat menisectomy | 4,507 | 1.83 |
| 58571 | Llaparoscopicaroscopy w tot hysterectuterus <=250 gram w tube/ovary | 4,295 | 1.75 |
| 27130 | Total hip arthroplasty | 4,229 | 1.72 |
| 49320 | Llaparoscopic, diagnostic abdomen | 4,069 | 1.66 |
| 44970 | Llaparoscopic, appendectomy | 3,587 | 1.46 |
| 43775 | Llaparoscopic, gast restrict procedure longitudinal gastrectomy | 3,302 | 1.34 |
| 52332 | Cystoscopy, insert ureteral stent | 3,234 | 1.32 |
| 52356 | Cysto/uretero w/lithotripsy &indwell stent insrt | 3,169 | 1.29 |
| 27447 | Total knee arthroplasty | 3,094 | 1.26 |
| 29827 | Shoulder arthroscop, surg, w/rotat cuff repr | 3,038 | 1.24 |
| 52000 | Cystourethroscopy | 2,925 | 1.19 |
| 32666 | Thoracoscopy w/thera wedge resexn initial unilat | 2,595 | 1.06 |
| 29888 | Knee scope, aid ant cruciate repair | 2,542 | 1.03 |
| 58662 | Llaparoscopic, fulgurate/excise lesions | 2,334 | 0.95 |
| 19303 | Mastectomy, simple, complete | 2,285 | 0.93 |
| 49000 | Exploratory of abdomen | 2,029 | 0.83 |
| 63047 | Laminec/facetect/foramin, lumbar 1 seg | 1,993 | 0.81 |
| 58558 | Hysteroscopy, w/endo bx | 1,983 | 0.81 |
| 20680 | Removal deep implant | 1,935 | 0.79 |
| 29877 | Knee scope, shave articular cart | 1,923 | 0.78 |
| 29826 | Shoulder arthroscop, part acromioplas | 1,905 | 0.77 |
| 32663 | Thoracoscopy surg lobectomy | 1,849 | 0.75 |

# Supplementary Table 3. Most frequent ICD-10 codes in the retrospective cohort.

| ICD | Description | n | Frequency (%) |
| --- | --- | --- | --- |
| I10 | Essential (primary) hypertension | 78,112 | 31.77 |
| E78.5 | Hyperlipidemia, unspecified | 44,035 | 17.91 |
| K21.9 | Gastro-esophageal reflux disease without esophagitis | 32,952 | 13.40 |
| Z00.00 | Encounter for general adult medical examination without abnormal findings | 31,061 | 12.63 |
| F32.A | Depression, unspecified | 22,271 | 9.06 |
| F41.9 | Anxiety disorder, unspecified | 21,591 | 8.78 |
| E78.00 | Pure hypercholesterolemia, unspecified | 20,086 | 8.17 |
| E11.9 | Type 2 diabetes mellitus without complications | 19,461 | 7.92 |
| E03.9 | Hypothyroidism, unspecified | 18,765 | 7.63 |
| J45.909 | Unspecified asthma, uncomplicated | 18,671 | 7.59 |
| E66.9 | Obesity, unspecified | 18,428 | 7.50 |
| M54.50 | Low back pain, unspecified | 15,950 | 6.49 |
| D64.9 | Anemia, unspecified | 15,229 | 6.19 |
| M19.90 | Unspecified osteoarthritis, unspecified site | 15,096 | 6.14 |
| G89.29 | Other chronic pain | 14,928 | 6.07 |
| I25.10 | Atherosclerotic heart disease of native coronary artery without angina pectoris | 14,872 | 6.05 |
| E55.9 | Vitamin D deficiency, unspecified | 14,633 | 5.95 |
| Z98.890 | Other specified postprocedural states | 13,829 | 5.62 |
| M81.0 | Age-related osteoporosis without current pathological fracture | 11,503 | 4.68 |
| N20.0 | Calculus of kidney | 11,168 | 4.54 |
| G47.33 | Obstructive sleep apnea (adult) (pediatric) | 10,591 | 4.31 |
| R10.9 | Unspecified abdominal pain | 10,174 | 4.14 |
| G43.909 | Migraine, unspecified, not intractable, without status migrainosus | 10,116 | 4.11 |
| M25.569 | Pain in unspecified knee | 9,458 | 3.85 |
| F17.200 | Nicotine dependence, unspecified, uncomplicated | 9,354 | 3.80 |

# Supplementary Table 4. Most frequent CPT codes in the prospective cohort.

| CPT | Description | n | Frequency (%) |
| --- | --- | --- | --- |
| 63047 | Laminec/facetect/foramin, lumbar 1 seg | 14 | 3.83 |
| 44207 | Llaparoscopic, surg, colectomy, w/anast | 13 | 3.55 |
| 64713 | Neuroplasty brachial plexus, open | 12 | 3.28 |
| 21615 | Removal 1st/cervical rib | 12 | 3.28 |
| 43775 | Llaparoscopic, gast restrict procedure longitudinal gastrectomy | 12 | 3.28 |
| 55840 | Remv prostate, retropub, radical | 11 | 3.01 |
| 32663 | Thoracoscopy surg lobectomy | 11 | 3.01 |
| 31622 | Brnchsc incl fluor gdnce dx w/cell washg spx | 11 | 3.01 |
| 22630 | Arthrodesis posterior interbody lumbar | 8 | 2.19 |
| 48150 | Part remv panc, prox+remv duod+anast | 8 | 2.19 |
| 23472 | Reconstr total shoulder implant | 8 | 2.19 |
| 61510 | Excis supratent brain tumor | 7 | 1.91 |
| 49000 | Exploratory of abdomen | 7 | 1.91 |
| 50547 | Llaparoscopic, donor kidney remov, living | 6 | 1.64 |
| 44160 | Remvl colon & term ileum w/ileocolostomy | 6 | 1.64 |
| 44205 | Llaparoscopic, surg, colectomy, w/remvl term ileum | 6 | 1.64 |
| 63051 | C-laminoplasty w/graft/plate, 2 or more | 5 | 1.37 |
| 49203 | Excision/destruction open abdominal tumors 5 cm | 5 | 1.37 |
| 60650 | Llaparoscopic, adrenalectomy | 5 | 1.37 |
| 50360 | Transplantation of kidney | 5 | 1.37 |
| 32484 | Removal of lung, segmentectomy | 5 | 1.37 |
| 50543 | Llaparoscopic, partial nephrectomy | 5 | 1.37 |
| 55866 | Llaparoscopic, prostatectomy, radical, w/nerve spare, incl robotic | 5 | 1.37 |
| 47999 | Bile tract surg procedure unlisted | 4 | 1.09 |
| 96549 | Chemotherapy, unspecified procedure | 4 | 1.09 |

# Supplementary Table 5. Most frequent ICD-10 codes in the prospective cohort.

| ICD | Description | n | Frequency (%) |
| --- | --- | --- | --- |
| I10 | Essential (primary) hypertension | 119 | 32.51 |
| Z00.00 | Encounter for general adult medical examination without abnormal findings | 87 | 23.77 |
| E78.5 | Hyperlipidemia, unspecified | 71 | 19.40 |
| K21.9 | Gastro-esophageal reflux disease without esophagitis | 53 | 14.48 |
| M54.50 | Low back pain, unspecified | 44 | 12.02 |
| F41.9 | Anxiety disorder, unspecified | 42 | 11.48 |
| G89.29 | Other chronic pain | 39 | 10.66 |
| E55.9 | Vitamin D deficiency, unspecified | 34 | 9.29 |
| E03.9 | Hypothyroidism, unspecified | 33 | 9.02 |
| E78.00 | Pure hypercholesterolemia, unspecified | 31 | 8.47 |
| I25.10 | Atherosclerotic heart disease of native coronary artery without angina pectoris | 30 | 8.20 |
| Z98.890 | Other specified postprocedural states | 29 | 7.92 |
| E11.9 | Type 2 diabetes mellitus without complications | 29 | 7.92 |
| M54.2 | Cervicalgia | 28 | 7.65 |
| M19.90 | Unspecified osteoarthritis, unspecified site | 28 | 7.65 |
| F32.A | Depression, unspecified | 28 | 7.65 |
| R91.1 | Solitary pulmonary nodule | 26 | 7.10 |
| D64.9 | Anemia, unspecified | 26 | 7.10 |
| E66.9 | Obesity, unspecified | 25 | 6.83 |
| E66.01 | Morbid (severe) obesity due to excess calories | 23 | 6.28 |
| Z01.818 | Encounter for other preprocedural examination | 22 | 6.01 |
| R10.9 | Unspecified abdominal pain | 22 | 6.01 |
| J45.909 | Unspecified asthma, uncomplicated | 22 | 6.01 |
| G47.33 | Obstructive sleep apnea (adult) (pediatric) | 21 | 5.74 |
| E78.2 | Mixed hyperlipidemia | 19 | 5.19 |

# Supplementary Table 6. Baseline statistics for the subgroup of patients with outcomes on postoperative day 0. MGH: Massachusetts General Hospital, BWH: Brigham and Women’s Hospital, NSMC: North Shore Medical Center, NWH: Newton Wellesley Hospital, ASA: American Society of Anesthesiologist Performance Status.

| Statistic | Retrospective | (%) | Prospective | (%) |
| --- | --- | --- | --- | --- |
| Total patients | 217,096 | (100.0) | 344 | (100.00) |
| MGH | 104,224 | (48.01) | 344 | (100.00) |
| BWH | 71,669 | (33.01) | 0 | (0.00) |
| NSMC | 28,843 | (13.29) | 0 | (0.00) |
| NWH | 12,360 | (5.69) | 0 | (0.00) |
| Demographics |  |  |  |  |
| Age, mean (SD) years | 55.7 | (17.0) | 59.5 | (16.0) |
| Gender |  |  |  |  |
| Male | 94,739 | (43.64) | 171 | (49.71) |
| Female | 122,351 | (56.36) | 173 | (50.29) |
| Race |  |  |  |  |
| White | 178,595 | (82.27) | 279 | (81.10) |
| Black | 10,836 | (4.99) | 12 | (3.49) |
| Hispanic | 8,338 | (3.84) | 19 | (5.52) |
| Asian | 6,630 | (3.05) | 9 | (2.62) |
| Other | 12,697 | (5.85) | 25 | (7.27) |
| Height, mean (SD) m | 1.69 | (0.11) | 1.69 | (0.12) |
| Weight, mean (SD) kg | 81.48 | (24.69) | 83.30 | (23.00) |
| Opioid naivety | 152,115 | (70.07) | 226 | (65.70) |
| Clinical characteristics |  |  |  |  |
| ASA |  |  |  |  |
| I | 22,054 | (10.16) | 10 | (2.91) |
| II | 109,113 | (50.26) | 127 | (36.92) |
| III | 81,522 | (37.55) | 200 | (58.14) |
| IV | 4,194 | (1.93) | 7 | (2.03) |
| Ambulatory surgery | 90,232 | (41.56) | 0 | (0.00) |
| Inpatient surgery | 126,860 | (58.43) | 344 | (100.00) |
| Elixhauser comorbidity index, median (IQR) | 0.0 | (0.0, 5.0) | 0.0 | (0.0, 5.0) |
| Surgical service |  |  |  |  |
| Orthopedic Surgery | 53,783 | (24.77) | 60 | (17.44) |
| General Surgery | 33,993 | (15.66) | 103 | (29.94) |
| Urology | 23,283 | (10.72) | 32 | (9.30) |
| Gynecology | 21,465 | (9.89) | 9 | (2.62) |
| Thoracic Surgery | 14,872 | (6.85) | 49 | (14.24) |
| Neurosurgery | 12,654 | (5.83) | 54 | (15.70) |
| Surgical Oncology | 13,281 | (6.12) | 0 | (0.00) |
| Other | 43,765 | (20.16) | 37 | (10.76) |

# Supplementary Table 7. Baseline statistics for the subgroup of patients with outcomes on postoperative day 1. MGH: Massachusetts General Hospital, BWH: Brigham and Women’s Hospital, NSMC: North Shore Medical Center, NWH: Newton Wellesley Hospital, ASA: American Society of Anesthesiologist Performance Status.

| Statistic | Retrospective | (%) | Prospective | (%) |
| --- | --- | --- | --- | --- |
| Total patients | 158,200 | (100.0) | 350 | (100.00) |
| MGH | 57,743 | (36.50) | 350 | (100.00) |
| BWH | 67,861 | (42.90) | 0 | (0.00) |
| NSMC | 24,162 | (15.27) | 0 | (0.00) |
| NWH | 8,434 | (5.33) | 0 | (0.00) |
| Demographics |  |  |  |  |
| Age, mean (SD) years | 58.1 | (16.6) | 59.8 | (16.0) |
| Gender |  |  |  |  |
| Male | 71,339 | (45.09) | 175 | (50.00) |
| Female | 86,855 | (54.90) | 175 | (50.00) |
| Race |  |  |  |  |
| White | 130,865 | (82.72) | 284 | (81.14) |
| Black | 8,334 | (5.27) | 13 | (3.71) |
| Hispanic | 6,595 | (4.17) | 20 | (5.71) |
| Asian | 4,441 | (2.81) | 9 | (2.57) |
| Other | 7,965 | (5.03) | 24 | (6.86) |
| Height, mean (SD) m | 1.69 | (0.11) | 1.69 | (0.13) |
| Weight, mean (SD) kg | 82.04 | (23.69) | 83.15 | (23.11) |
| Opioid naivety | 108,849 | (68.80) | 225 | (64.29) |
| Clinical characteristics |  |  |  |  |
| ASA |  |  |  |  |
| I | 10,186 | (6.44) | 9 | (2.57) |
| II | 68,228 | (43.13) | 124 | (35.43) |
| III | 73,519 | (46.47) | 209 | (59.71) |
| IV | 5,975 | (3.78) | 7 | (2.00) |
| Ambulatory surgery | 20,872 | (13.19) | 0 | (0.00) |
| Inpatient surgery | 137,327 | (86.81) | 350 | (100.00) |
| Elixhauser comorbidity index, median (IQR) | 1.0 | (0.0, 7.0) | 0.0 | (0.0, 5.0) |
| Surgical service |  |  |  |  |
| Orthopedic Surgery | 38,699 | (24.46) | 59 | (16.86) |
| General Surgery | 24,183 | (15.29) | 97 | (27.71) |
| Urology | 15,291 | (9.67) | 32 | (9.14) |
| Gynecology | 11,114 | (7.03) | 10 | (2.86) |
| Thoracic Surgery | 13,812 | (8.73) | 54 | (15.43) |
| Neurosurgery | 15,433 | (9.76) | 62 | (17.71) |
| Surgical Oncology | 8,545 | (5.40) | 0 | (0.00) |
| Other | 31,123 | (19.67) | 36 | (10.29) |

# Supplementary Table 8. Baseline statistics for the subgroup of patients with outcomes on postoperative day 2. MGH: Massachusetts General Hospital, BWH: Brigham and Women’s Hospital, NSMC: North Shore Medical Center, NWH: Newton Wellesley Hospital, ASA: American Society of Anesthesiologist Performance Status.

| Statistic | Retrospective | (%) | Prospective | (%) |
| --- | --- | --- | --- | --- |
| Total patients | 96,364 | (100.0) | 264 | (100.00) |
| MGH | 36,306 | (37.68) | 264 | (100.00) |
| BWH | 45,707 | (47.43) | 0 | (0.00) |
| NSMC | 9,131 | (9.48) | 0 | (0.00) |
| NWH | 5,220 | (5.42) | 0 | (0.00) |
| Demographics |  |  |  |  |
| Age, mean (SD) years | 60.3 | (16.4) | 60.7 | (15.9) |
| Gender |  |  |  |  |
| Male | 44,894 | (46.59) | 129 | (48.86) |
| Female | 51,464 | (53.41) | 135 | (51.14) |
| Race |  |  |  |  |
| White | 79,806 | (82.82) | 211 | (79.92) |
| Black | 5,432 | (5.64) | 12 | (4.55) |
| Hispanic | 3,788 | (3.93) | 16 | (6.06) |
| Asian | 2,527 | (2.62) | 7 | (2.65) |
| Other | 4,811 | (4.99) | 18 | (6.82) |
| Height, mean (SD) m | 1.69 | (0.11) | 1.69 | (0.13) |
| Weight, mean (SD) kg | 81.86 | (25.03) | 80.76 | (23.53) |
| Opioid naivety | 63,502 | (65.90) | 162 | (61.36) |
| Clinical characteristics |  |  |  |  |
| ASA |  |  |  |  |
| I | 2,888 | (3.00) | 8 | (3.03) |
| II | 33,572 | (34.84) | 82 | (31.06) |
| III | 54,288 | (56.34) | 168 | (63.64) |
| IV | 5,373 | (5.58) | 5 | (1.89) |
| Ambulatory surgery | 1,854 | (1.92) | 0 | (0.00) |
| Inpatient surgery | 94,510 | (98.08) | 264 | (100.00) |
| Elixhauser comorbidity index, median (IQR) | 3.0 | (0.0, 9.0) | 0.0 | (0.0, 5.0) |
| Surgical service |  |  |  |  |
| Orthopedic Surgery | 25,597 | (26.56) | 42 | (15.91) |
| General Surgery | 14,309 | (14.85) | 72 | (27.27) |
| Urology | 6,177 | (6.41) | 10 | (3.79) |
| Gynecology | 3,935 | (4.08) | 10 | (3.79) |
| Thoracic Surgery | 10,592 | (10.99) | 46 | (17.42) |
| Neurosurgery | 11,549 | (11.98) | 55 | (20.83) |
| Surgical Oncology | 4,549 | (4.72) | 0 | (0.00) |
| Other | 19,656 | (20.40) | 29 | (10.98) |

# Supplementary Table 9. Baseline statistics for the subgroup of patients with outcomes on postoperative day 3. MGH: Massachusetts General Hospital, BWH: Brigham and Women’s Hospital, NSMC: North Shore Medical Center, NWH: Newton Wellesley Hospital, ASA: American Society of Anesthesiologist Performance Status.

| Statistic | Retrospective | (%) | Prospective | (%) |
| --- | --- | --- | --- | --- |
| Total patients | 70,216 | (100.0) | 192 | (100.00) |
| MGH | 26,478 | (37.71) | 192 | (100.00) |
| BWH | 34,297 | (48.84) | 0 | (0.00) |
| NSMC | 5,734 | (8.17) | 0 | (0.00) |
| NWH | 3,707 | (5.28) | 0 | (0.00) |
| Demographics |  |  |  |  |
| Age, mean (SD) years | 61.2 | (16.5) | 62.3 | (14.9) |
| Gender |  |  |  |  |
| Male | 33,503 | (47.71) | 96 | (50.00) |
| Female | 36,707 | (52.28) | 96 | (50.00) |
| Race |  |  |  |  |
| White | 58,107 | (82.75) | 154 | (80.21) |
| Black | 4,077 | (5.81) | 10 | (5.21) |
| Hispanic | 2,639 | (3.76) | 10 | (5.21) |
| Asian | 1,868 | (2.66) | 3 | (1.56) |
| Other | 3,525 | (5.02) | 15 | (7.81) |
| Height, mean (SD) m | 1.69 | (0.11) | 1.68 | (0.14) |
| Weight, mean (SD) kg | 80.89 | (25.57) | 80.82 | (24.90) |
| Opioid naivety | 45,078 | (64.20) | 110 | (57.29) |
| Clinical characteristics |  |  |  |  |
| ASA |  |  |  |  |
| I | 1,852 | (2.64) | 4 | (2.08) |
| II | 21,462 | (30.57) | 50 | (26.04) |
| III | 41,825 | (59.57) | 132 | (68.75) |
| IV | 4,866 | (6.93) | 5 | (2.60) |
| Ambulatory surgery | 2,526 | (3.60) | 0 | (0.00) |
| Inpatient surgery | 67,690 | (96.40) | 192 | (100.00) |
| Elixhauser comorbidity index, median (IQR) | 4.0 | (0.0, 9.0) | 1.0 | (0.0, 5.0) |
| Surgical service |  |  |  |  |
| Orthopedic Surgery | 19,076 | (27.17) | 26 | (13.54) |
| General Surgery | 9,110 | (12.97) | 62 | (32.29) |
| Urology | 3,940 | (5.61) | 8 | (4.17) |
| Gynecology | 2,668 | (3.80) | 7 | (3.65) |
| Thoracic Surgery | 7,848 | (11.18) | 26 | (13.54) |
| Neurosurgery | 8,108 | (11.55) | 41 | (21.35) |
| Surgical Oncology | 3,604 | (5.13) | 0 | (0.00) |
| Other | 15,862 | (22.59) | 22 | (11.46) |

# Supplementary Table 10. Baseline statistics for the subgroup of patients with outcomes on postoperative day 4. MGH: Massachusetts General Hospital, BWH: Brigham and Women’s Hospital, NSMC: North Shore Medical Center, NWH: Newton Wellesley Hospital, ASA: American Society of Anesthesiologist Performance Status.

| Statistic | Retrospective | (%) | Prospective | (%) |
| --- | --- | --- | --- | --- |
| Total patients | 50,326 | (100.0) | 140 | (100.00) |
| MGH | 19,310 | (38.37) | 140 | (100.00) |
| BWH | 25,342 | (50.36) | 0 | (0.00) |
| NSMC | 3,052 | (6.06) | 0 | (0.00) |
| NWH | 2,622 | (5.21) | 0 | (0.00) |
| Demographics |  |  |  |  |
| Age, mean (SD) years | 61.3 | (16.5) | 63.2 | (14.6) |
| Gender |  |  |  |  |
| Male | 25,233 | (50.14) | 73 | (52.14) |
| Female | 25,087 | (49.85) | 67 | (47.86) |
| Race |  |  |  |  |
| White | 41,214 | (81.89) | 115 | (82.14) |
| Black | 3,128 | (6.22) | 7 | (5.00) |
| Hispanic | 1,974 | (3.92) | 7 | (5.00) |
| Asian | 1,381 | (2.74) | 2 | (1.43) |
| Other | 2,629 | (5.22) | 9 | (6.43) |
| Height, mean (SD) m | 1.69 | (0.11) | 1.68 | (0.15) |
| Weight, mean (SD) kg | 80.69 | (22.23) | 81.07 | (26.76) |
| Opioid naivety | 31,532 | (62.66) | 81 | (57.86) |
| Clinical characteristics |  |  |  |  |
| ASA |  |  |  |  |
| I | 1,009 | (2.00) | 4 | (2.86) |
| II | 13,236 | (26.30) | 26 | (18.57) |
| III | 31,539 | (62.67) | 106 | (75.71) |
| IV | 4,355 | (8.65) | 3 | (2.14) |
| Ambulatory surgery | 1,442 | (2.87) | 0 | (0.00) |
| Inpatient surgery | 48,884 | (97.13) | 140 | (100.00) |
| Elixhauser comorbidity index, median (IQR) | 4.0 | (0.0, 10.0) | 2.0 | (0.0, 7.0) |
| Surgical service |  |  |  |  |
| Orthopedic Surgery | 11,708 | (23.26) | 15 | (10.71) |
| General Surgery | 6,552 | (13.02) | 53 | (37.86) |
| Urology | 2,970 | (5.90) | 7 | (5.00) |
| Gynecology | 1,554 | (3.09) | 5 | (3.57) |
| Thoracic Surgery | 6,036 | (11.99) | 19 | (13.57) |
| Neurosurgery | 5,714 | (11.35) | 27 | (19.29) |
| Surgical Oncology | 3,007 | (5.98) | 0 | (0.00) |
| Other | 12,785 | (25.40) | 14 | (10.00) |

# Supplementary Table 11. AUC by surgical service for moderate pain (max pain >4) in the retrospective cohort

| Service | Postop Day 0 | Postop Day 1 | Postop Day 2 | Postop Day 3 | Postop Day 4 |
| --- | --- | --- | --- | --- | --- |
| Orthopedic Surgery | 0.788 (0.775, 0.800) | 0.800 (0.784, 0.815) | 0.724 (0.701, 0.746) | 0.726 (0.703, 0.750) | 0.718 (0.688, 0.747) |
| General Surgery | 0.766 (0.749, 0.782) | 0.740 (0.720, 0.761) | 0.696 (0.669, 0.724) | 0.694 (0.659, 0.729) | 0.722 (0.683, 0.761) |
| Urology | 0.723 (0.703, 0.744) | 0.773 (0.750, 0.796) | 0.721 (0.681, 0.761) | 0.687 (0.635, 0.739) | 0.684 (0.622, 0.747) |
| Gynecology | 0.699 (0.677, 0.722) | 0.811 (0.785, 0.836) | 0.683 (0.631, 0.734) | 0.689 (0.630, 0.748) | 0.737 (0.664, 0.810) |
| Thoracic Surgery | 0.814 (0.791, 0.837) | 0.767 (0.740, 0.794) | 0.697 (0.664, 0.731) | 0.701 (0.664, 0.738) | 0.718 (0.677, 0.759) |
| Neurosurgery | 0.749 (0.718, 0.779) | 0.733 (0.706, 0.760) | 0.725 (0.694, 0.755) | 0.730 (0.695, 0.765) | 0.750 (0.710, 0.790) |
| Surgical Oncology | 0.724 (0.697, 0.751) | 0.769 (0.738, 0.800) | 0.707 (0.660, 0.755) | 0.704 (0.652, 0.757) | 0.715 (0.659, 0.770) |
| Plastic Surgery | 0.678 (0.647, 0.710) | 0.794 (0.760, 0.827) | 0.729 (0.672, 0.785) | 0.749 (0.687, 0.811) | 0.743 (0.673, 0.812) |
| Otolaryngology | 0.705 (0.663, 0.747) | 0.824 (0.780, 0.867) | 0.751 (0.674, 0.828) | 0.828 (0.757, 0.899) | 0.777 (0.677, 0.877) |
| Vascular Surgery | 0.737 (0.694, 0.779) | 0.772 (0.734, 0.810) | 0.771 (0.728, 0.813) | 0.776 (0.728, 0.824) | 0.782 (0.729, 0.835) |

# Supplementary Table 12. AUC by surgical service for severe pain (max pain >6) in the retrospective cohort

| Service | Postop Day 0 | Postop Day 1 | Postop Day 2 | Postop Day 3 | Postop Day 4 |
| --- | --- | --- | --- | --- | --- |
| Orthopedic Surgery | 0.772 (0.760, 0.785) | 0.756 (0.741, 0.771) | 0.698 (0.677, 0.718) | 0.717 (0.694, 0.740) | 0.697 (0.667, 0.727) |
| General Surgery | 0.741 (0.725, 0.758) | 0.728 (0.707, 0.748) | 0.721 (0.693, 0.749) | 0.722 (0.686, 0.758) | 0.729 (0.688, 0.769) |
| Urology | 0.692 (0.667, 0.717) | 0.785 (0.758, 0.812) | 0.712 (0.667, 0.758) | 0.691 (0.632, 0.750) | 0.681 (0.610, 0.753) |
| Gynecology | 0.677 (0.653, 0.701) | 0.788 (0.756, 0.820) | 0.738 (0.686, 0.790) | 0.733 (0.668, 0.798) | 0.763 (0.679, 0.847) |
| Thoracic Surgery | 0.790 (0.767, 0.813) | 0.740 (0.714, 0.767) | 0.677 (0.643, 0.712) | 0.692 (0.651, 0.733) | 0.709 (0.663, 0.755) |
| Neurosurgery | 0.742 (0.715, 0.769) | 0.704 (0.678, 0.730) | 0.712 (0.682, 0.742) | 0.716 (0.679, 0.752) | 0.727 (0.684, 0.770) |
| Surgical Oncology | 0.725 (0.698, 0.753) | 0.738 (0.704, 0.771) | 0.701 (0.650, 0.752) | 0.704 (0.646, 0.762) | 0.686 (0.624, 0.748) |
| Plastic Surgery | 0.660 (0.629, 0.690) | 0.772 (0.737, 0.807) | 0.740 (0.685, 0.794) | 0.747 (0.687, 0.808) | 0.775 (0.709, 0.841) |
| Otolaryngology | 0.733 (0.688, 0.778) | 0.807 (0.757, 0.858) | 0.764 (0.685, 0.842) | 0.791 (0.709, 0.873) | 0.816 (0.723, 0.910) |
| Vascular Surgery | 0.763 (0.723, 0.802) | 0.800 (0.766, 0.835) | 0.796 (0.755, 0.837) | 0.785 (0.737, 0.832) | 0.764 (0.711, 0.818) |

# Supplementary Table 13. RMSE by surgical service in the retrospective cohort

| Service | Postop Day 0 | Postop Day 1 | Postop Day 2 | Postop Day 3 | Postop Day 4 |
| --- | --- | --- | --- | --- | --- |
| Orthopedic Surgery | 2.687 (2.640, 2.736) | 2.233 (2.182, 2.283) | 2.190 (2.131, 2.250) | 2.292 (2.225, 2.361) | 2.409 (2.318, 2.503) |
| General Surgery | 2.535 (2.482, 2.591) | 2.320 (2.256, 2.388) | 2.375 (2.293, 2.457) | 2.517 (2.409, 2.633) | 2.621 (2.486, 2.757) |
| Urology | 2.850 (2.780, 2.916) | 2.534 (2.450, 2.617) | 2.710 (2.566, 2.849) | 2.866 (2.680, 3.056) | 2.926 (2.707, 3.145) |
| Gynecology | 2.447 (2.381, 2.515) | 2.146 (2.052, 2.240) | 2.447 (2.279, 2.609) | 2.418 (2.232, 2.611) | 2.508 (2.262, 2.748) |
| Thoracic Surgery | 2.479 (2.393, 2.572) | 2.441 (2.354, 2.532) | 2.538 (2.436, 2.636) | 2.591 (2.467, 2.703) | 2.731 (2.584, 2.868) |
| Neurosurgery | 2.598 (2.496, 2.700) | 2.526 (2.447, 2.607) | 2.541 (2.450, 2.631) | 2.694 (2.587, 2.807) | 2.741 (2.597, 2.875) |
| Surgical Oncology | 2.604 (2.517, 2.691) | 2.345 (2.238, 2.449) | 2.307 (2.164, 2.450) | 2.312 (2.153, 2.475) | 2.368 (2.212, 2.521) |
| Plastic Surgery | 2.533 (2.434, 2.631) | 2.180 (2.065, 2.299) | 2.420 (2.260, 2.580) | 2.572 (2.358, 2.772) | 2.629 (2.396, 2.856) |
| Otolaryngology | 2.569 (2.436, 2.700) | 2.317 (2.126, 2.518) | 2.829 (2.524, 3.148) | 2.585 (2.275, 2.887) | 2.969 (2.572, 3.361) |
| Vascular Surgery | 2.829 (2.677, 2.986) | 2.896 (2.751, 3.047) | 2.773 (2.616, 2.922) | 2.831 (2.653, 2.989) | 2.715 (2.528, 2.917) |

# Supplementary Table 14. Correlation by surgical service in the retrospective cohort

| Service | Postop Day 0 | Postop Day 1 | Postop Day 2 | Postop Day 3 | Postop Day 4 |
| --- | --- | --- | --- | --- | --- |
| Orthopedic Surgery | 0.588 (0.569, 0.606) | 0.587 (0.565, 0.609) | 0.414 (0.381, 0.446) | 0.454 (0.418, 0.489) | 0.425 (0.379, 0.470) |
| General Surgery | 0.515 (0.489, 0.540) | 0.500 (0.467, 0.531) | 0.430 (0.386, 0.472) | 0.431 (0.377, 0.482) | 0.424 (0.356, 0.486) |
| Urology | 0.441 (0.407, 0.475) | 0.553 (0.517, 0.588) | 0.457 (0.389, 0.520) | 0.377 (0.281, 0.463) | 0.374 (0.262, 0.484) |
| Gynecology | 0.406 (0.367, 0.444) | 0.573 (0.531, 0.612) | 0.415 (0.322, 0.500) | 0.454 (0.352, 0.544) | 0.471 (0.340, 0.593) |
| Thoracic Surgery | 0.605 (0.571, 0.638) | 0.573 (0.533, 0.608) | 0.418 (0.356, 0.471) | 0.434 (0.373, 0.494) | 0.451 (0.372, 0.523) |
| Neurosurgery | 0.477 (0.430, 0.523) | 0.442 (0.403, 0.479) | 0.474 (0.429, 0.517) | 0.486 (0.434, 0.538) | 0.507 (0.444, 0.568) |
| Surgical Oncology | 0.466 (0.423, 0.512) | 0.526 (0.478, 0.573) | 0.384 (0.298, 0.465) | 0.393 (0.302, 0.485) | 0.414 (0.315, 0.505) |
| Plastic Surgery | 0.370 (0.319, 0.419) | 0.600 (0.552, 0.644) | 0.518 (0.432, 0.596) | 0.521 (0.420, 0.615) | 0.536 (0.427, 0.633) |
| Otolaryngology | 0.467 (0.401, 0.532) | 0.629 (0.550, 0.696) | 0.414 (0.244, 0.557) | 0.595 (0.440, 0.718) | 0.470 (0.262, 0.646) |
| Vascular Surgery | 0.478 (0.414, 0.538) | 0.523 (0.469, 0.576) | 0.550 (0.486, 0.610) | 0.549 (0.483, 0.612) | 0.558 (0.478, 0.627) |

# Supplementary Table 15. AUC in the retrospective cohort for moderate pain (max pain >4). MGH: Massachusetts General Hospital, BWH: Brigham and Women’s Hospital, NSMC: North Shore Medical Center, NWH: Newton Wellesley Hospital.

| Training site | Day 0 | Day 1 | Day 2 | Day 3 | Day 4 |
| --- | --- | --- | --- | --- | --- |
| All | 0.763 | 0.792 | 0.739 | 0.732 | 0.739 |
| MGH | 0.739 | 0.804 | 0.730 | 0.729 | 0.725 |
| BWH | 0.769 | 0.793 | 0.756 | 0.736 | 0.752 |
| NWH | 0.763 | 0.776 | 0.735 | 0.730 | 0.741 |
| NSMC | 0.765 | 0.794 | 0.739 | 0.732 | 0.740 |

# Supplementary Table 16. AUC in the retrospective cohort for severe pain (max pain >6). MGH: Massachusetts General Hospital, BWH: Brigham and Women’s Hospital, NSMC: North Shore Medical Center, NWH: Newton Wellesley Hospital.

| Training site | Day 0 | Day 1 | Day 2 | Day 3 | Day 6 |
| --- | --- | --- | --- | --- | --- |
| All | 0.751 | 0.769 | 0.728 | 0.736 | 0.735 |
| MGH | 0.732 | 0.774 | 0.722 | 0.733 | 0.730 |
| BWH | 0.756 | 0.777 | 0.737 | 0.740 | 0.740 |
| NWH | 0.752 | 0.756 | 0.726 | 0.737 | 0.735 |
| NSMC | 0.752 | 0.772 | 0.727 | 0.735 | 0.734 |

# Supplementary Table 17. RMSE in the retrospective cohort. MGH: Massachusetts General Hospital, BWH: Brigham and Women’s Hospital, NSMC: North Shore Medical Center, NWH: Newton Wellesley Hospital.

| Training site | Day 0 | Day 1 | Day 2 | Day 3 | Day 4 |
| --- | --- | --- | --- | --- | --- |
| All | 2.630 | 2.389 | 2.443 | 2.538 | 2.618 |
| MGH | 2.752 | 2.550 | 2.536 | 2.631 | 2.723 |
| BWH | 2.973 | 2.513 | 2.517 | 2.610 | 2.669 |
| NWH | 2.797 | 2.606 | 2.662 | 2.699 | 2.796 |
| NSMC | 2.904 | 2.649 | 2.602 | 2.704 | 2.796 |

# Supplementary Table 18. Correlation in the retrospective cohort. MGH: Massachusetts General Hospital, BWH: Brigham and Women’s Hospital, NSMC: North Shore Medical Center, NWH: Newton Wellesley Hospital.

| Training site | Day 0 | Day 1 | Day 2 | Day 3 | Day 4 |
| --- | --- | --- | --- | --- | --- |
| All | 0.536 | 0.581 | 0.486 | 0.484 | 0.486 |
| MGH | 0.438 | 0.505 | 0.419 | 0.438 | 0.435 |
| BWH | 0.446 | 0.525 | 0.442 | 0.432 | 0.450 |
| NWH | 0.455 | 0.480 | 0.370 | 0.391 | 0.378 |
| NSMC | 0.398 | 0.439 | 0.369 | 0.364 | 0.365 |

# Supplementary Table 19. O/E ratio for moderate pain (max pain >4) with 95% confidence intervals.

| Day | Retrospective | Prospective | Clinician |
| --- | --- | --- | --- |
| 0 | 0.979 (0.976, 0.981) | 1.080 (1.027, 1.135) | 1.075 (1.000, 1.158) |
| 1 | 1.006 (1.003, 1.009) | 1.120 (1.060, 1.179) | 1.341 (1.224, 1.477) |
| 2 | 0.980 (0.976, 0.984) | 1.085 (1.013, 1.165) | 1.540 (1.348, 1.800) |
| 3 | 0.977 (0.973, 0.982) | 1.067 (0.973, 1.159) | 2.203 (1.812, 2.791) |
| 4 | 0.998 (0.992, 1.005) | 1.029 (0.906, 1.149) | 2.351 (1.786, 3.185) |

# Supplementary Table 20. O/E ratio for severe pain (max pain >6) with 95% confidence intervals.

| Day | Retrospective | Prospective | Clinician |
| --- | --- | --- | --- |
| 0 | 0.944 (0.939, 0.948) | 1.216 (1.116, 1.327) | 1.383 (1.205, 1.608) |
| 1 | 0.977 (0.972, 0.983) | 1.184 (1.076, 1.305) | 2.000 (1.654, 2.468) |
| 2 | 0.989 (0.982, 0.995) | 1.178 (1.044, 1.317) | 3.559 (2.631, 5.167) |
| 3 | 1.008 (0.999, 1.017) | 1.143 (0.977, 1.323) | 6.167 (3.941, 12.429) |
| 4 | 1.033 (1.022, 1.043) | 1.215 (1.016, 1.427) | 9.667 (5.182, 32.500) |

# Supplementary Table 21. Calibration intercept, with 95% confidence intervals.

| Day | Retrospective | Prospective | Clinician |
| --- | --- | --- | --- |
| 0 | -0.314 (-0.350, -0.279) | 2.435 (1.088, 3.781) | 5.212 (4.475, 5.949) |
| 1 | -0.113 (-0.150, -0.077) | 2.763 (1.828, 3.699) | 4.729 (4.053, 5.405) |
| 2 | -0.348 (-0.410, -0.286) | 1.215 (0.158, 2.273) | 4.836 (4.079, 5.592) |
| 3 | -0.166 (-0.231, -0.101) | 0.406 (-0.851, 1.663) | 4.278 (3.374, 5.182) |
| 4 | -0.213 (-0.290, -0.135) | 0.111 (-1.408, 1.629) | 4.509 (3.482, 5.536) |

# Supplementary Table 22. Calibration slope, with 95% confidence intervals

| Day | Retrospective | Prospective | Clinician |
| --- | --- | --- | --- |
| 0 | 1.045 (1.038, 1.051) | 0.720 (0.489, 0.950) | 0.232 (0.114, 0.350) |
| 1 | 1.003 (0.996, 1.009) | 0.635 (0.475, 0.795) | 0.321 (0.198, 0.444) |
| 2 | 1.057 (1.046, 1.068) | 0.909 (0.716, 1.101) | 0.282 (0.123, 0.440) |
| 3 | 1.066 (1.054, 1.078) | 1.093 (0.843, 1.342) | 0.393 (0.174, 0.613) |
| 4 | 1.025 (1.011, 1.039) | 1.121 (0.817, 1.425) | 0.296 (0.026, 0.566) |

# Supplementary Table 23. AUC in the prospective cohort for moderate pain (max pain >4). MGH: Massachusetts General Hospital, BWH: Brigham and Women’s Hospital, NSMC: North Shore Medical Center, NWH: Newton Wellesley Hospital.

| Predictor | Day 0 | Day 1 | Day 2 | Day 3 | Day 4 |
| --- | --- | --- | --- | --- | --- |
| Clinician | 0.660 | 0.665 | 0.595 | 0.636 | 0.589 |
| All | 0.687 | 0.689 | 0.707 | 0.739 | 0.729 |
| MGH | 0.688 | 0.671 | 0.686 | 0.742 | 0.764 |
| BWH | 0.646 | 0.670 | 0.696 | 0.689 | 0.709 |
| NSMC | 0.648 | 0.687 | 0.671 | 0.686 | 0.740 |
| NWH | 0.603 | 0.669 | 0.641 | 0.695 | 0.743 |

# Supplementary Table 24. AUC in the prospective cohort for severe pain (max pain >6). MGH: Massachusetts General Hospital, BWH: Brigham and Women’s Hospital, NSMC: North Shore Medical Center, NWH: Newton Wellesley Hospital.

| Predictor | Day 0 | Day 1 | Day 2 | Day 3 | Day 4 |
| --- | --- | --- | --- | --- | --- |
| Clinician | 0.621 | 0.625 | 0.580 | 0.609 | 0.627 |
| All | 0.673 | 0.675 | 0.753 | 0.794 | 0.782 |
| MGH | 0.708 | 0.663 | 0.764 | 0.809 | 0.774 |
| BWH | 0.663 | 0.661 | 0.728 | 0.729 | 0.714 |
| NSMC | 0.644 | 0.663 | 0.739 | 0.760 | 0.758 |
| NWH | 0.652 | 0.672 | 0.735 | 0.746 | 0.769 |

# Supplementary Table 25. RMSE in the prospective cohort. MGH: Massachusetts General Hospital, BWH: Brigham and Women’s Hospital, NSMC: North Shore Medical Center, NWH: Newton Wellesley Hospital.

| Predictor | Day 0 | Day 1 | Day 2 | Day 3 | Day 4 |
| --- | --- | --- | --- | --- | --- |
| Clinician | 3.214 | 2.869 | 3.216 | 3.396 | 3.563 |
| All | 2.533 | 2.231 | 2.186 | 2.324 | 2.268 |
| MGH | 2.647 | 2.310 | 2.257 | 2.412 | 2.392 |
| BWH | 2.522 | 2.178 | 2.246 | 2.443 | 2.461 |
| NSMC | 2.575 | 2.300 | 2.383 | 2.405 | 2.363 |
| NWH | 2.717 | 2.398 | 2.466 | 2.388 | 2.307 |

# Supplementary Table 26. Correlation in the prospective cohort. MGH: Massachusetts General Hospital, BWH: Brigham and Women’s Hospital, NSMC: North Shore Medical Center, NWH: Newton Wellesley Hospital.

| Predictor | Day 0 | Day 1 | Day 2 | Day 3 | Day 4 |
| --- | --- | --- | --- | --- | --- |
| Clinician | 0.204 | 0.265 | 0.211 | 0.250 | 0.181 |
| All | 0.311 | 0.372 | 0.494 | 0.537 | 0.541 |
| MGH | 0.314 | 0.386 | 0.497 | 0.529 | 0.524 |
| BWH | 0.302 | 0.386 | 0.458 | 0.452 | 0.423 |
| NSMC | 0.296 | 0.371 | 0.369 | 0.433 | 0.506 |
| NWH | 0.270 | 0.410 | 0.469 | 0.496 | 0.549 |
