# Supplementary material for: Development and prospective validation of postoperative pain prediction from preoperative EHR data using attention-based set embeddings
Source: NPJ Digit Med. 2023 Nov 16;6:209. doi: 10.1038/s41746-023-00947-z (PMC10654400; doi:10.1038/s41746-023-00947-z)
Supplement: Supplementary file 2 — Reporting Summary [file 41746_2023_947_MOESM2_ESM.pdf]

nature portfolio

Corresponding author(s): Patrick L. Purdon

Last updated by author(s): Sep 4, 2023

Reporting Summary

Nature Portfolio wishes to improve the reproducibility of the work that we publish. This form provides structure for consistency and transparency in reporting. For further information on Nature Portfolio policies, see our Editorial Policies and the Editorial Policy Checklist.

Statistics

For all statistical analyses, confirm that the following items are present in the figure legend, table legend, main text, or Methods section.

n/a	Confirmed	
☐	☒	The exact sample size (*n*) for each experimental group/condition, given as a discrete number and unit of measurement
☐	☒	A statement on whether measurements were taken from distinct samples or whether the same sample was measured repeatedly
☐	☒	The statistical test(s) used AND whether they are one- or two-sided<br>*Only common tests should be described solely by name; describe more complex techniques in the Methods section.*
☐	☒	A description of all covariates tested
☐	☒	A description of any assumptions or corrections, such as tests of normality and adjustment for multiple comparisons
☐	☒	A full description of the statistical parameters including central tendency (e.g. means) or other basic estimates (e.g. regression coefficient) AND variation (e.g. standard deviation) or associated estimates of uncertainty (e.g. confidence intervals)
☐	☒	For null hypothesis testing, the test statistic (e.g. *F*, *t*, *r*) with confidence intervals, effect sizes, degrees of freedom and *P* value noted<br>*Give P values as exact values whenever suitable.*
☒	☐	For Bayesian analysis, information on the choice of priors and Markov chain Monte Carlo settings
☒	☐	For hierarchical and complex designs, identification of the appropriate level for tests and full reporting of outcomes
☐	☒	Estimates of effect sizes (e.g. Cohen's *d*, Pearson's *r*), indicating how they were calculated

Our web collection on statistics for biologists contains articles on many of the points above.

Software and code

Policy information about availability of computer code

Data collection	Electronic health record data was extracted using SQL queries from the Mass General Brigham Institutional Enterprise Data Warehouse system.
Data analysis	Data analysis was performed using PyTorch version 1.13.1 and R version 4.2.3. Figures were created using ggplot2. Tables were created using flextable.

For manuscripts utilizing custom algorithms or software that are central to the research but not yet described in published literature, software must be made available to editors and reviewers. We strongly encourage code deposition in a community repository (e.g. GitHub). See the Nature Portfolio guidelines for submitting code & software for further information.

Data

Policy information about availability of data

All manuscripts must include a data availability statement. This statement should provide the following information, where applicable:

- Accession codes, unique identifiers, or web links for publicly available datasets
- A description of any restrictions on data availability
- For clinical datasets or third party data, please ensure that the statement adheres to our policy

Access to data used in this study requires a Data Use Agreement and IRB approval by the study institutions (MGB). Contingent upon these requirements, data are available from the authors upon reasonable request.

Research involving human participants, their data, or biological material

Policy information about studies with human participants or human data. See also policy information about sex, gender (identity/presentation), and sexual orientation and race, ethnicity and racism.

Reporting on sex and gender	Patient sex was determined based on how these variables were recorded in the electronic health record (EPIC). In our study, sex is included as a covariate in our prediction model.
Reporting on race, ethnicity, or other socially relevant groupings	Race and ethnicity were determined based on how these variables were recorded in the electronic health record (EPIC), and were grouped based on categories stated in the NIH notice NOT-OD-15-089 (https://grants.nih.gov/grants/guide/notice-files/not-od-15-089.html). In our study, race is included as a covariate in our prediction model.
Population characteristics	Our study was conducted on EHR data from 234,274 adult patients who underwent non-cardiac surgery with general anesthesia. Of the patients in our retrospective study cohort, 130,713 (55.79%) were women; 192,664 (82.24%) were White non-Hispanic. The mean age was 55.9 years (SD 17.0). Orthopedic, general, urological, gynecological, and thoracic surgeries were the most common, comprising a combined 66.8% of surgeries.
Recruitment	For prospective section we enrolled all consecutive patients that met our inclusion criteria. For retrospective part there is no recruitment
Ethics oversight	The protocol for the retrospective component of this study and a waiver of informed consent for participants were approved by the Massachusetts General Hospital (MGH) institutional review board (IRB #2020P000301). Our prospective validation protocol was also approved by the MGH IRB (#2022P002958).

Note that full information on the approval of the study protocol must also be provided in the manuscript.

Field-specific reporting

Please select the one below that is the best fit for your research. If you are not sure, read the appropriate sections before making your selection.

☒ Life sciences ☐ Behavioural & social sciences ☐ Ecological, evolutionary & environmental sciences

For a reference copy of the document with all sections, see nature.com/documents/nr-reporting-summary-flat.pdf

Life sciences study design

All studies must disclose on these points even when the disclosure is negative.

Sample size	Power calculations were performed using G*Power 3.1.9.7 to estimate the minimum number of patients required for our prospective study to identify significant differences in predictive performance between clinicians and our model. We computed the required sample size and parameters estimated from the empirical residual distribution of our model-based predictions of postoperative pain in the retrospective cohort. To estimate the residual distribution of clinician predictions, we computed the residual distribution for predicting the population mean pain for every patient. For a power of 0.90 and an α of 0.05 in a paired signed-rank test of performance between our model and clinician predictions, we estimated a required sample size of 365 patients.
Data exclusions	We excluded subjects in whom no outcome was reported.
Replication	Multicenter development of the model. Validation using a fraction of the sample and prospective validation
Randomization	NA
Blinding	NA

Reporting for specific materials, systems and methods

We require information from authors about some types of materials, experimental systems and methods used in many studies. Here, indicate whether each material, system or method listed is relevant to your study. If you are not sure if a list item applies to your research, read the appropriate section before selecting a response.

Materials & experimental systems

n/a	Involved in the study	
☒	☐	Antibodies
☒	☐	Eukaryotic cell lines
☒	☐	Palaeontology and archaeology
☒	☐	Animals and other organisms
☐	☒	Clinical data
☒	☐	Dual use research of concern
☒	☐	Plants

Methods

n/a	Involved in the study	
☒	☐	ChIP-seq
☒	☐	Flow cytometry
☒	☐	MRI-based neuroimaging

Clinical data

Policy information about clinical studies

All manuscripts should comply with the ICMJE guidelines for publication of clinical research and a completed CONSORT checklist must be included with all submissions.

Clinical trial registration
NA

Study protocol
The protocol for the retrospective component of this study and a waiver of informed consent for participants were approved by the Massachusetts General Hospital (MGH) institutional review board (IRB #2020P000301). Our prospective validation protocol was also approved by the MGH IRB (#2022P002958). Study protocols are detailed in Supplementary Methods.

Data collection
Retrospective part: two quaternary care academic medical centers, MGH and BWH, and two community hospitals, NSMC and NWH, between April 1st, 2016, and March 31st, 2020. Our prospective study included adult patients who underwent inpatient non-cardiac surgery with general anesthesia at MGH between February 15th, 2023, and March 20th, 2023.

Outcomes
The outcome studied were the maximal post-operative pain scores reported by patients on the day of the surgery (Postoperative Day 0), and on the four subsequent days (Postoperative Day 1 through 4). Pain is generally assessed using the Numeric Rating Scale (NRS)53, although in some cases is reported as strings. In these cases, we converted strings into numeric variables using 6 categories (“no pain” - 0, “mild pain” - 2, “moderate pain” - 4, “severe pain” - 6, “very severe pain” - 8, and “worst possible pain” - 10). If multiple pain scores were recorded on a given day for a single patient, the highest value was kept. Since pain scores were extracted directly from the EHR, no blinding was required.
